# Proceedings of the 24^th^ Paediatric Rheumatology European Society Congress: Part three: Athens, Greece. 14-17 September 2017

**DOI:** 10.1186/s12969-017-0187-8

**Published:** 2017-09-01

**Authors:** Erato Atsali, Dimitra Kassara, Pelagia Katsimbri, Sorina Boiu, Dimitrios T. Boumpas, Vana Papaevangelou, Olena A. Oshlyanska, Ludmila I. Omelchenko, Tatiana A. Ljudvik, Katerina Bouchalova, Marcel Schüller, Jana Franova, Jarmila Skotakova, Marie Macku, Antoni Fellas, Fiona Hawke, Derek Santos, Andrea Coda, Anthi Kelempisioti, Paula Keskitalo, Virpi Glumoff, Petri Kulmala, Paula Vahasalo, Mohammed A. Mozaffar, Asraa K. Turkistani, Samaa O. Sangoof, Vladislav Sevostyanov, Elena Zholobova, Evangelia Bountouvi, konstantinos Theodoropoulos, Erato Atsali, Renata Moutsiou, Christina Tsalapaki, Pelagia Katsimbri, Sorina Boiu, Dimitrios T. Boumpas, Vana Papaevangelou, Talia Diaz, Sofia Osorio, Maria Teresa Braña, Yuridiana Ramirez, Luis Aparicio, Andres Rodriguez, Enrique Faugier, Rocio Maldonado, Maria Francesca Gicchino, Carmela Granato, Giulia Macchini, Daniela Capalbo, Alma Nunzia Olivieri, Nathan Hasson, Achille Marino, Sona Narula, Melissa Lerman, Maria Amelia Muñoz Calonge, Sara Maria Murias Loza, Rosa Maria Alcobendas, Agustin Remesal, Esmeralda Núñez-Cuadros, Rocio Galindo Zavala, Gisela Díaz-Cordovés Rego, Cristina Antúnez Fernández, Yaiza García Molina, Antonio L. Urda Cardona, Nihal Sahin, Habibe S. Durmus, Ayse S. Pinarbasi, Zubeyde Gunduz, Muammer H. Poyrazoglu, Zehra F. Karaman, Turhan Oktem, Mithat Oner, Ruhan Dusunsel, Gordana Susic, Tamara Krstajic, Dragana Vujovic, Nedeljko Radlovic, Zoran Lekovic, Dusica Novakovic, Gordana Milosevski Lomic, Karina Mördrup, Gunilla Hesselstrand, Iva Sorić, Lovro Lamot, Mandica Vidovic, Mirta Lamot, Miroslav Harjacek, Eva Adank, Elvira Cannizzaro Schneider, Eiman Abdalla, Irfan Ullah, L. Jeyaseelan, Reem Abdwani, Reem Abdwani, Laila A. L. Shaqsi, Ibrahim A. l. Zakwani, Erato Atsali, Pelagia Katsimbri, Antonis Fanouriakis, Sorina Boiu, Vana Papaevangelou, Dimitrios T. Boumpas, Mahesh Janarthanan, Dhanarathnamoorthy Vetrichelvan, P. Ramachandran, Sangeetha Geminiganesan, Dinesh Kumar, Subba Rao, Eleni-Maria Papatesta, Despoina Maritsi, Irini Eleftheriou, Maria Tsolia, Olga Vougiouka, Mustafa Çakan, Nuray Aktay Ayaz, Şerife Gül Karadağ, Gonca Keskindemirci, Vladimir Keltsev, Lyudmila Grebenkina, Kwang Nam Kim, Jong Gyun Ahn, Young Dae Kim, Maria Cristina Maggio, Rolando Cimaz, Maria Concetta Failla, Piera Dones, Mirella Collura, Giovanni Corsello, Jung-Woo Rhim, Ki-Hwan Kim, Soo-Young Lee, Seung-Beom Han, Jin-Han Kang, Jae-Hee Chung, Soo-Jung Lee, Dae-Chul Jeong, Andrei Santimov, Regina Rupp, Igor Alekseev, Natalya Plutova, Ekaterina Moskvina, Marina Kruchina, Aleksandra Tarasenko, Natalya Sokolova, Ekaterina Saveleva, Ilia Bogdanov, Dmitrii Ivanov, Tatiana Kandrina, Olga Kopanevich, Anastasiia Grafskaia, Natalia Ignateva, Daria Pulukchu, Natalia Pavlova, Olga Kalashnikova, Tatiana Kornishina, Margarita Dubko, Vyacheslav Chasnyk, Mikhail Kostik, Shama Sowdagar, Janani Sankar, Venkateswari Ramesh, Mahesh Janarthanan, Iulia E. Szabo, Claudia Sirbe, Cristina Pamfil, Laura Damian, Simona Rednic, Maria Deac, Cristina Pamfil, Mihaela Sparchez, Ileana Filipescu, Mirela Parvu, Dumitrita Balint, Ancuta Nicoara, Simona Rednic, Laura Damian

**Affiliations:** 1 Pediatric Rheumatology Unit, 3RD Department of Pediatrics, National and Kapodestrian University of Athens, Medical School, Athens, Greece; 2 Joint Rheumatology Program, 4TH Department of Medicine, National and Kapodestrian University of Athens, Medical School, Athens, Greece; 3 Department of connective tissue disorders in children, State Institute of Pediatrics, Obstetrics and Gynecology, Academy of Medical Sciences of Ukraine, Kyiv, Ukraine; 4 Pediatric Rheumatology Unit, Pediatric Department, University Hospital Brno, Brno, Czech Republic; 5 Department of Pediatrics, Palacky University, Olomouc, Czech Republic; 6 Institute of Molecular and Translational Medicine, Palacky University, Olomouc, Czech Republic; 7 Pediatric Radiology Department, University Hospital Brno, Brno, Czech Republic; 8 School of Health Sciences, Faculty of Health and Medicine, University of Newcastle, Newcastle, Australia; 9 School of Health Sciences, Queen Margaret University, Edinburgh, UK; 10 Medical Immunology and Microbiology, University of Oulu, Oulu, Finland; 11 Pediatrics, University of Oulu, Oulu, Finland; 12 Pediatric, King Abdulaziz University Hospital, Facult Of Medicine, Jeddah, Saudi Arabia; 13 Municipal Children’s Polyclinic № 122, Moscow, Russian Federation; 14 I.M. Sechenov First Moscow State Medical University, Moscow, Russian Federation; 15 Pediatric Rheumatology Unit, 3RD Department of Pediatrics, National and Kapodistrian University of Athens, Medical School, Athens, Greece; 16 2nd Department of Dermatology and Venereology, National and Kapodistrian University of Athens, Medical School, Athens, Greece; 17 Clinical Immunology-Rheumatology Unit, 2nd Department of Medicine and Laboratory, National and Kapodistrian University of Athens, Medical School, Athens, Greece; 18 Joint Rheumatology Program, 4TH Department of Medicine, National and Kapodistrian University of Athens, Medical School, Athens, Greece; 19 Pediatric Rheumatology, Hospital Infantil de Mexico Federico Gomez, Ciudad de Mexico, Mexico; 20 Department of Women, Child and General and Specialistic Surgery, Università della Campania Luigi Vanvitelli, Naples, Italy; 21 Paediatric Rheumatology, The Portland Hospital, London, UK; 22 Rheumatology, Meyer Children’s Hospital, Florence, Italy; 23 Neurology, The Children’s Hospital of Philadelphia, Philadelphia, PA USA; 24 Rheumatology, The Children’s Hospital of Philadelphia, Philadelphia, PA USA; 25 Pediatric Rheumatology, Pediatrician, Madrid, Spain; 26 Paediatric Rheumatology, Universitary Regional Malaga Hospital, Malaga, Spain; 27 Paediatrics, Universitary Regional Malaga Hospital, Malaga, Spain; 28 Pediatric Rheumatology, Erciyes University Faculty of Medicine, Kayseri, Turkey; 29 Pediatrics, Erciyes University Faculty of Medicine, Kayseri, Turkey; 30 Pediatric Nephrology, Erciyes University Faculty of Medicine, Kayseri, Turkey; 31 Radiology, Erciyes University Faculty of Medicine, Kayseri, Turkey; 32 Pathology, Erciyes University Faculty of Medicine, Kayseri, Turkey; 33 Ortopedics, Erciyes University Faculty of Medicine, Kayseri, Turkey; 34 Institute of Rheumatology, Belgrade, Serbia; 35 University Children’s hospital, Belgrade, Serbia; 36 School of Medicine, University of Belgrade, Belgrade, Serbia; 37 Pediatric Rheumatology Unit, Karolinska University Hospital, Stockholm, Sweden; 38 Pediatric Orthopedic Unit, Karolinska University Hospital, Stockholm, Sweden; 39 Clinical Hospital Center Sestre Milosrdnice, Zagreb, Croatia; 40 Univeristy of Zagreb School of Medicine, Zagreb, Croatia; 41 Department of Paediatric Rheumatology, Kinderspital Zürich, Zürich, Switzerland; 42 Child health, Sultan Qaboos University Hospital, Muscat, Oman; 43 Biostatistics, Sultan Qaboos University Hospital, Muscat, Oman; 44 Child Health, Sultan Qaboos University, Muscat, Oman; 45 Child Health, Oman Medical Speciality Board, Muscat, Oman; 46 Pediatric Rheumatology Unit, 3RD Department of Pediatrics, National and Kapodestrian University of Athens, Medical School, Athens, Greece; 47 Joint Rheumatology Program, 4TH Department of Medicine, National and Kapodestrian University of Athens, Medical School, Athens, Greece; 48 Division of Pediatric Reumatology, Sri Ramachandra University, Chennai, India; 49 Department of Pediatrics, Sri Ramachandra University, Chennai, India; 50 Second department of Pediatrics, P. & A. Kyriakou Children’s Hospital, Athens, Greece; 51 Pediatric Rheumatology, Kanuni Sultan Süleyman Research And Training Hospital, İstanbul, Turkey; 52 Pediatrics, Kanuni Sultan Süleyman Research And Training Hospital, İstanbul, Turkey; 53 Rheumatology, Togliatti City Clinical Hospital #5, Togliatti, Russian Federation; 54 Pediatrics, Hallym University Medical Center, AnYang, Korea, Republic Of; 55 Pediatrics, Severance Children’s Hospital, Yonsei University, Seoul, Korea, Republic Of; 56 Pediatrics, Inje University Paik Hospital, KoYang, Korea, Republic Of; 57 University Department Pro.Sa.M.I. “G. D’Alessandro”, University of Palermo, Palermo, Italy; 58 Pediatric Rheumatology Unit, Neurofarba Department, AOU Meyer, University of Florence, Florence, Italy; 59 Paediatric Infectious Disease Unit, Children Hospital “G. Di Cristina”, ARNAS Palermo, Palermo, Italy; 60 Unit of Paediatric Pneumology and Cystic Fibrosis, Children Hospital “G. Di Cristina”, ARNAS Palermo, Palermo, Italy; 61 Pediatrics, College of Medicine, The Catholic University of Korea, Seoul, Korea, Republic Of; 62 General Surgery, College of Medicine, The Catholic University of Korea, Seoul, Korea, Republic Of; 63 Pediatrics, International St. Mary’s Hospital, Catholic Kwandong University, Incheon, Korea, Republic Of; 64 Saint-Petersburg State Pediatric Medical University, Saint-Petersburg, Russian Federation; 65 Saint Petersburg Children’s Hospital №1, Saint-Petersburg, Russian Federation; 66 Department of Pediatrics, Kanchi Kamakoti Childs Trust Hospital, Chennai, India; 67 Consultant Pediatric Rheumatologist, Kanchi Kamakoti Childs Trust Hospital, Chennai, India; 68 Department of Rheumatology, Emergency Clinical County Hospital, Cluj-Napoca, Romania; 69 Department of Pediatrics, Emergency Clinical County Hospital, Cluj-Napoca, Romania; 70 Department of Rheumatology, “Iuliu Hatieganu” University of Medicine and Pharmacy, Cluj-Napoca, Romania; 71 Emergency Clinical County Hospital, Cluj-Napoca, Romania; 72 Rheumatology, Iuliu Hatieganu University of Medicine and Pharmacy, Cluj-Napoca, Romania; 73 Centre for Rare Musculoskeletal Autoimmune and Autoinflammatory Diseases, Cluj-Napoca, Romania; 74 Pediatrics, Iuliu Hatieganu University of Medicine and Pharmacy, Cluj-Napoca, Romania; 75 Rheumatology, Emergency Clinical County Hospital, Targu-Mures, Romania; 76 Emergency Clinical County Hospital, Targu-Mures, Romania; 77 Centre for Rare Musculoskeletal Autoimmune and Autoinflammatory Diseases, Cluj-Napoca, Romania; 78 Rheumatology, Centre for Rare Musculoskeletal Autoimmune and Autoinflammatory Diseases, Cluj-Napoca, Romania

## Autoinflammatory diseases

### B1 Periodic fever, aphthous stomatitis, pharyngitis, and adenopathy syndrome (PFAPA) in a 17 years old girl – case report

#### Erato Atsali^1^, Dimitra Kassara^2^, Pelagia Katsimbri^2^, Sorina Boiu^1^, Dimitrios T. Boumpas^2^, Vana Papaevangelou^1^

##### ^1^Pediatric Rheumatology Unit,3RD Department of Pediatrics, National and Kapodestrian University of Athens, Medical School, Athens, Greece; ^2^Joint Rheumatology Program,4TH Department of Medicine, National and Kapodestrian University of Athens, Medical School, Athens, Greece

###### **Correspondence:** Erato Atsali

**Introduction:** Periodic fever, aphthous stomatitis, pharyngitis, and cervical adenopathy (PFAPA) syndrome is a non Mendelian autoinflammatory disorder until now considered to be specifically limited to paediatric age. However there is recently mounting evidence that children older than 5 years and adults may present with the typical picture of PFAPA syndrome.

**Objectives:** We report the case of a 17,5 years old girl presenting with recurrent episodes of fever starting at the age of 16.

**Methods:** A 17,5 years old girl was referred to our department due to recurrent episodes of fever lasting 3–4 days and starting 1,5 year ago. Body temperature usually increased up to 40.5∘C. Fever was accompanied by exudative pharyngitis, cervical lympadenopathy, severe malaise and myalgias/artralgias. Oral apthosis was present in some of the episodes. There was no other sign of upper respiratory tract infection. Headache and fatigue were evident 2–3 days before each flare.The episodes recurred very regularly showing an almost clockwork periodicity (every 35–40 days). The girl was completely asymptomatic between flares with normal growth and development. The girl’s mother suffered from similar episodes of exudative pharyngitis until approximately the age of 12.

**Results:** Laboratory exams during febrile attacks showed mild leukocytosis with prominence of neutrophils and a significant increase of monocytes. Erythrocyte sedimentation rate,C-reactive protein and Serum Amyloid A were also raised. Serum immunoglobulin levels (including IgD) were normal. During episodes all the cultures were negative.Inflammatory markers were completely normal between attacks.The girl was diagnosed with PFAPA syndrome based on Marshall criteria revised by Thomas et al.,neglecting the item of disease onset before the age of five. Genetic testing for FMF is pending. Tonsillectomy was not proposed as a therapeutic option because it seems not to be effective in adults, based on current literature. A single dose of prednisone (1 mg/kg) given a few hours after the onset of fever dramatically abrupted fever attack in a few hours, supporting the diagnosis of PFAPA.The girl did not experience a free interval shortening after steroid administration.

**Conclusion:** Once thought to be exclusive of the pediatric age, it is nowadays clear that PFAPA syndrome may have its onset in adulthood. Clinical features of PFAPA adults are overlapping with those of PFAPA children.To date, it is not known whether adults with PFAPA syndrome may spontaneously undergo clinical remission, and tonsillectomy does not seem to be a valid option in these patients. The description of PFAPA syndrome in adults should increase awareness among clinicians and suggests that the age criterion (i.e., age at onset less than 5 years) should not be considered mandatory for diagnosis

**Disclosure of Interest**

None Declared

## Disease outcome

### B2 Case of paraneoplastic arthritis in child

#### Olena A. Oshlyanska, Ludmila I. Omelchenko, Tatiana A. Ljudvik

##### Department of connective tissue disorders in children, State Institute of Pediatrics, Obstetrics and Gynecology, Academy of Medical Sciences of Ukraine, Kyiv, Ukraine

###### **Correspondence:** Olena A. Oshlyanska

**Introduction:** Differential diagnosis in children’s articular syndrome presents difficulties in actual practice. According to the clinical register of the connective tissue separation diseases in children more than 20% of cases with a primary diagnosis of JIA are overdiagnosis. Special attention should be paid to arthritis in neoplastic processes, they are characterized by the injury of one joint with severe pain syndrome and hematological changes.

**Objectives:** A description of the case of paraneoplastic arthritis.

**Methods:** Analysis of medical history

**Results:** Child Ts., A boy of 14 years. Anamnesis is not burdensome. For 3 months before hospitalization - an acute intestinal infection, after 3 weeks - pain and swelling of the right knee joint, subfebrilititis, NSAID therapy 1 month without effect. On examination, swelling without local hyperthermia of the right knee joint with minimal restriction of flexion, “crunching” during movement, other joints are intact. Dryness of the skin on the back surface of the hands, moderate difficulty in nasal breathing. At physical examination no changes in internal organs revealed. At examination, the general blood test without pathological changes, ESR 10 mm/h, CRP negative. RF, ANA, APLAT, aDNA - not detected, serological tests for pseudotuberculosis, iersiniosis, mycoplasmal and chlamydial infections are negative, ASLO is normal. Elevated CIC 0.059 g/l, IgE in serum (934 ng/ml), positive skin prik test for histamine, eosinophilia in the nazocytogram and peripheral blood. ECG, ultrasound examination - without changes. Ultrasound of the knee joint: exudative synovitis. Ultrasound of the thyroid gland: left-sided nodular goiter (node 30x15x17x5.8 mm). T3, T4, TSH, ATPO are normal. A thin needle aspiration needle biopsy of the thyroid gland was performed. Cytological study: pronounced proliferation and atypia of thyroid epithelium. Left-sided hemithyroidectomy was performed. By the conclusion of a biopsy “papillary carcinoma of the thyroid gland”. The child was examined 1.5 months after the operation. Clinical manifestations of arthritis of knee joints have not been revealed. NSAIDs are canceled, receiving substitution therapy with thyroxine. There was swelling of the face and a one-sided enlargement of the cervical lymph nodes, with their biopsy atypical cells that do not morphologically correspond to the cells of the thyroid gland identified. The CT of the whole organism was carried out - the primary localization of the tumor was not established. The patient is currently observed at the oncologist.

**Conclusion:** In some cases, arthritis develops earlier than the tumor process is diagnosed. The clinician should pay attention to the lack of typical clinical and laboratory signs characteristic of JIA and resistance to anti-inflammatory therapy. Manifestations of allergization, like arthritis, may be signs of a rheumatological paraneoplastic syndrome.

**Disclosure of Interest**

None Declared

## Juvenile idiopathic arthritis (oligo, poly, psoriatic)

### B3 A patient with three diagnoses? juvenile idiopathic arthritis, chronic recurrent multifocal osteomyelitis and atypical subacute Moraxella osloensis infection

#### Katerina Bouchalova^1,2,3^, Marcel Schüller^1^, Jana Franova^1^, Jarmila Skotakova^4^, Marie Macku^1^

##### ^1^Pediatric Rheumatology Unit, Pediatric Department, University Hospital Brno, Brno, Czech Republic; ^2^Department of Pediatrics, Palacky University, Olomouc, Olomouc, Czech Republic; ^3^Institute of Molecular and Translational Medicine, Palacky University, Olomouc, Olomouc, Czech Republic; ^4^Pediatric Radiology Department, University Hospital Brno, Brno, Czech Republic

###### **Correspondence:** Katerina Bouchalova

**Introduction:** Chronic recurrent multifocal osteomyelitis (CRMO) as a rare autoinflammatory disorder, affecting predominantly girls, is sometimes associated with arthritis.

**Objectives:** Report of a patient with subsequent rheumatology diagnoses.

**Methods:** A case report of a 10-year-old girl with juvenile idiopathic arthritis (JIA) with onset at the age of 1 year and 8 months, in remission on therapy (etanercept + methotrexate) presenting with new clinical problems leading to subsequent diagnoses.

**Results:** At the age of 10 years, the girl presented with backpain lasting 2 weeks, swelling localized to thoracic region, elevated ESR, CRP, and anemia. Whole body MRI revealed Th8/Th9 compressive fracture and multifocal bone lesions. Clindamycine and Jewett corset were recommended with discontinuation of immunosuppressive therapies. Lesional biopsy, bone marrow aspiration and PET-CT did not show malignancy. When intermittent fever appeared, blood culture revealed *Moraxella osloensis* (confirmed by PCR) resistant to clindamycine and antibiotics were changed and administered up to 14.5 weeks in total. Despite the slow clinical improvement the interdisciplinary discussions led to initiation of bisphosphonate therapy. On intravenous pamidronate administered every 3 months (now after the third course), her clinical status as well as imaging findings are steadily improving, with no signs of arthritis.

**Conclusion:** Discussion: The following questions arise: Was it “only” an atypical infection in an immunocompromised patient with JIA or does the patient suffer from three rare diseases (JIA, subacute osteomyelitis by *Moraxella osloensis*, and CRMO)?

Parents agreed with presentation of patient’case, including photodocumentation.

**Disclosure of Interest**

None Declared

### B4 Foot and ankle pathologies in juvenile idiopathic arthritis: a narrative review

#### Antoni Fellas^1^, Fiona Hawke^1^, Derek Santos^2^, Andrea Coda^1^

##### ^1^School of Health Sciences, Faculty of Health and Medicine, University of Newcastle, Newcastle, Australia; ^2^School of Health Sciences, Queen Margaret University, Edinburgh, United Kingdom

###### **Correspondence:** Antoni Fellas

**Introduction:** Foot and ankle pathologies are common in juvenile idiopathic arthritis (JIA) and can cause physical disability and reduce quality of life (1). Early detection and evidence-based treatment of these symptomatic pathologies are an important first step in preventing ongoing pain and long-term disabilities in children with JIA.

**Objectives:** To search the literature and provide an update on the types of foot and ankle pathologies reported in children with JIA.

**Methods:** MEDLINE (Ovid) was searched for relevant papers published in English with preference given to papers published in the last 10 years, and older highly regarded cited papers.

**Results:** Foot and ankle pathologies are highly prevalent in JIA (1–3). Foot and ankle pathologies in JIA include joint disease, tenosynovitis, muscle atrophy, enthesitis, digital deformities and biomechanical abnormalities (1–9). One study surveying foot problems found that in a cohort of 30 children with JIA, 63% reported some level of foot-related impairment and 60% with foot-related participation restriction (1). This review outlines and describes each of these foot and ankle pathologies.

*Joint disease –* Joint disease in JIA may include joint swelling, tenderness, pain, warmth and stiffness (4). These symptoms typically occur as a result of synovitis (4) and may be involved in 35-58% of cases (2, 3, 5, 10).

*Tenosynovitis –* inflammation of the tendon sheath in JIA commonly affects the tibialis posterior and peroneal tendons (5).

*Muscle atrophy –* Plantar-flexor muscle atrophy may be observed in children with JIA. This may be more noticeable when there is active joint disease in the ankle (6, 7). A reduction of plantar-flexion strength at the ankle may have implications in the propulsive phase of the gait, by delaying heel lift and increasing plantar pressures on the rear and midfoot.

*Enthesitis –* Inflammation at the site of insertion of a tendon or ligament to the bone is common at the Achilles tendon and the medial tubercle of the calcaneus. These are typically seen in male patients with the enthesitis-related subtype of JIA (4). One recent study with 26 JIA participants (average age of 11.6 years) reported a prevalence of 45% for the Achilles tendon and 20% for the plantar fascia (8). The mean recorded pain on a 100 mm visual analogue scale was 48 mm (8). Quality of life was not measured in this study; however, this level of pain may reduce physical and social well-being.

*Digital deformities –* Inflammation in the forefoot may lead to digital deformities such as clawed toes in children and adolescents with JIA. One study reported a prevalence of 17% in 144 participants (average age = 10.6) with JIA and hallux abducto valgus (2). Children with polyarticular subtype of JIA and those with a longer duration of disease were more likely to have hallux abducto valgus (2).

*Biomechanical abnormalities –* Biomechanical abnormalities of the foot and ankle are associated with prolonged synovitis (3). Synovitis can disrupt normal articulation of the rear and midfoot joints, and can contribute to an excessively pronated foot and abnormal plantar pressures (9). One study found that the prevalence of excessively pronated rear and midfoot joints in 144 JIA participants, was 73% and 72% respectively (2).

**Conclusion:** A range of foot and ankle pathologies are highly prevalent in JIA and contribute to physical morbidity. Allied health professionals may be involved as part of the paediatric rheumatology multidisciplinary team to assist in the early detection and management of these lower limb pathologies. Further research is required to attain accurate prevalence rates and the long-term implications that these foot and ankle conditions may have on a child or adolescent with JIA.

References

1. Hendry G, Gardner-Medwin J, Watt GF, Woodburn J. A survey of foot problems in juvenile idiopathic arthritis. Musculoskelet. 2008;6(4):221–32.

2. Spraul G, Koenning G. A descriptive study of foot problems in children with juvenile rheumatoid arthritis (JRA). Arthritis Care Res. 1994;7(3):144–50.

3. Ferrari J. A review of the foot deformities seen in juvenile chronic arthritis. The Foot. 1998;8(4):193–6.

4. Ravelli A, Martini A. Juvenile idiopathic arthritis. The Lancet. 2007;369(9563):767–78.

5. Javadi S, Kan JH, Orth RC, DeGuzman M. Wrist and ankle MRI of patients with juvenile idiopathic arthritis: identification of unsuspected multicompartmental tenosynovitis and arthritis. AJR Am J Roentgenol. 2014;202(2):413–7.

6. Saarinen J, Lehtonen K, Malkia E, Lahdenne P. Lower extremity isometric strength in children with juvenile idiopathic arthritis. Clin Exp Rheumatol. 2008;26(5):947–53.

7. Brostrom E, Nordlund MM, Cresswell AG. Plantar- and dorsiflexor strength in prepubertal girls with juvenile idiopathic arthritis. Arch Phys Med Rehabil. 2004;85(8):1224–30.

8. Jousse‐Joulin S, Breton S, Cangemi C, Fenoll B, Bressolette L, De Parscau L, et al. Ultrasonography for detecting enthesitis in juvenile idiopathic arthritis. Arthritis Care Res. 2011;63(6):849–55.

9. Haber L, Womack E, Zimmerman C, Hughes J. Clinical manifestations and treatment of the pediatric rheumatoid patient. Clinics in podiatric medicine and surgery. 2010;27(2):219–33.

10. Hemke R, Nusman CM, van der Heijde DM, Doria AS, Kuijpers TW, Maas M, et al. Frequency of joint involvement in juvenile idiopathic arthritis during a 5-year follow-up of newly diagnosed patients: implications for MR imaging as outcome measure. Rheumatol Int. 2015;35(2):351–7.

**Disclosure of Interest**

None Declared

### B5 Characterization of TCELLS in PBMCS of JIA patients in pre and post medication phase of treatment

#### Anthi Kelempisioti^1^, Paula Keskitalo^2^, Virpi Glumoff^1^, Petri Kulmala^1,2^, Paula Vahasalo^2^

##### ^1^Medical Immunology and Microbiology, University of Oulu, Oulu, Finland; ^2^Pediatrics, University of Oulu, Oulu, Finland

###### **Correspondence:** Anthi Kelempisioti

**Introduction:** Regulatory T cells (Treg) are essential regulators of self-tolerance and are crucial for maintaining immune homeostasis. FOXP3 transcription factor is considered as a specific marker and the main regulator for the differentiation and function of Treg cells. Juvenile Idiopathic Arthritis (JIA) is the most common rheumatic disease of childhood, and characterization of the specific subsets of Treg is essential to unravel their role in the pathogenesis of juvenile idiopathic arthritis (JIA).

**Objectives:** CD4^+^CD25^+^FOXP3^+^ Treg cells, are some of the key players in immunoregulation**.** This study was undertaken to investigate the phenotype of CD4+ T cells in PBMCs from JIA patients in two stages of clinical analyses, pre and post treatment and compare them to a matched set of healthy controls.

**Methods:** The phenotypes of T cells of PBMCs from JIA patients and matched controls were determined by flow cytometry. Treg suppression capacity was analyzed with a CFSE‐based T cell suppression assay. Statistical analyses within and between the groups was performed using SPSS.

**Results:** Treg number was quantified by analyzing CD4^+^CD25^+^FOXP3^+^ cells from peripheral blood samples. The proportion and suppression capacity of regulatory T cells were different between the cases and controls (pre-treatment cases vs controls *P = 0,006*, post-treatment cases vs controls *P = 0.001*). With just one third of the data analyzed there were slight quantitative differences in regulatory T cells between the case and control groups but were not statistically significant.

**Conclusion:** Results shown here are not conclusive since this is an ongoing study.

**Disclosure of Interest**

None Declared

### B6 A 10-year Saudi experience of using adalimumab in treating Juvenile Idiopathic Arthritis

#### Mohammed A. Mozaffar, Asraa K. Turkistani, Samaa O. Sangoof

##### Pediatric, King Abdulaziz University Hospital, Facult of Medicine, Jeddah, Saudi Arabia

###### **Correspondence:** Mohammed A. Mozaffar

**Introduction:** Traditionally, management of Juvenile Idiopathic Arthritis (JIA) involves use of non-steroidal anti-inflammatory drugs (NSAIDS) or disease-modifying anti-rheumatic drugs (DMARDs), such as methotrexate (MTX) or sulfasalazine; or steroids. However, in several cases, a low therapeutic response or important side effects are encountered. This study reports our experience in using adalimumab in JIA patients by assessing the efficacy and safety of this treatment in this category of patients.

**Objectives:** To reports our experience in using adalimumab in JIA patients by assessing the efficacy and safety of this treatment in this category of patients.

**Methods:** A retrospective study was conducted among 38 patients with JIA at the Pediatric Department, King Abdulaziz Univesrity Hospital, Jeddah, Saudi Arabia, in the period January 2005 - March 2016. Patients’ records were reviewed and relevant demographic and clinical data were collected. Data were analyzed using SPSS version 21 and represented using tables.

**Results:** The 38 patients were distributed as 11 (28.9%) males and 27 (71.1%); mean ± SD age was 11.91 ± 4.54 (range = 3 – 19) years. Mean ± SD (range) disease duration was 3.26 ± 2.52 (0–12) years and most frequent diagnoses included polyarticular rheumatoid factor (RF) negative form 12 (31.6%), followed by systemic and oligoarticular JIA with 9 (23.7%) cases each. Before adalimumab,fever was present in 13 (34.2%) cases, followed by rash in 8 (21.0%) cases; while 21 (55.3%) were asymptomatic. Thirty-one (81.6%) were in failure of MTX, 19 (50%) of steroids, 7 (18.4%) of NSAIDS and 3 (7.9%) had had intraarticular injections. Biologically, ANA, RF and anti-CCP were positive in 22 (57.9%), 8 (21.1%) and 4 (10.5%) of the cases, respectively. Uveitis was present in 11 (28.9%) of the patients. Analysis of adalimumab efficacy showed 10 (52.6%) cases of complete remission, 9 (23.7%) of partial remission and 9 (23.7%) other where treatment was discontinued. Major adverse effects included local pain (4 [10.5%]), new onset uveitis (1 [2.6%]) andrash (1 [2.6%]), responsible of 1case of treatment discontinuation. Predictors for complete remission on adalimumab were oligoarticular form (β = 3.450, p = 0.009) and negative RF (β = 2.381, p = 0.036); while predictors for nonresponse, whether complete or partial, were polyarticular form (β = −3.784, p = 0.005) and positive anti-CCP (β = −3.178, p = 0.021).

**Conclusion:** Adalimumab is an efficient and relatively safe alternative in the treatment of JIA with relatively high remission rates and lower rates of adverse effects. Further multicentre experiences are warranted to prove its efficacy and safety in the Saudi patients.

**Disclosure of Interest**

None Declared

### B7 Gene-engineering biological therapy in children with juvenile idiopathic arthritis in Moscow

#### Vladislav Sevostyanov^1^, Elena Zholobova^2^

##### ^1^Municipal Children’s Polyclinic № 122, Moscow, Russian Federation; ^2^I.M. Sechenov First Moscow State Medical University, Moscow, Russian Federation

###### **Correspondence:** Vladislav Sevostyanov

**Introduction:** In recent years, the problems of children’s rheumatology have become one of the most important areas of pediatrics. In the treatment of juvenile idiopathic arthritis (JIA), from the end of the 20th century, truly revolutionary progress is being made, thanks to the emergence of gene-engineering biological therapy.

**Objectives:** to analyze the structure of bDMARD and csDMARD therapy in patients with different JIA subtypes residing in Moscow.

**Methods:** The research included 262 patients residing in Moscow aged from 1 year up to 17 years with rheumatic diseases treated by bDMARDs. From them boys are 80 (30,5%), girls are 182 (69,5%), mean age is 10 years (3–17).

The following parameters was analysed: subtypes of JIA prevalence, structure of bDMARDs; concomitant csDMARDs therapy**.**

**Results:** In total there are 262 children receive bDMARDs in Moscow for 01.12.2016. Among them 123 (46,9%) suffer from RF(−) polyarthritis, 62 (23,7%) - oligoarthritis, 53 (20,2%) - systemic arthritis, 9,2% - other JIA forms.

2. Structure of bDMARDs in JIA.

TNF-inhibitors prevail over other biologics in children (etanercept and adalimumab – 71,8%). Of 262 treatments the most frequent bDMARDs are etanercept - 47%, and adalimumab– 24,8%.

19,9% of patients are treated with tocilizumab and 8,3% with abatacept.

3. Structure of concomitant csDMARDs in JIA

Most of children – 73,3% (192 patients from 262) receive a concomitant therapy by various medicines. Dominating majority of patients receive methotrexate (80,8% of all children receiving csDMARDs).

**Conclusion:** 1. TNF-inhibitors prevail in biologic therapy in children an age from 1 to 17 years with JIA, etanercept - 47%, adalimumab– 24,8%.

2. Majority of patients receive methotrexate as a concomitant therapy (80,8% of all children with JIA).

**Disclosure of Interest**

None Declared

## Juvenile dermatomyositis

### B8 Whole-Body Magnetic Resonance Imaging (WBMRI) revealing an extensive muscle involvement in a child presenting with Gottron’s papules and moderate muscle weakness– case report

#### Evangelia Bountouvi^1^, konstantinos Theodoropoulos^2^, Erato Atsali^1^, Renata Moutsiou^1^, Christina Tsalapaki^3^, Pelagia Katsimbri^4^, Sorina Boiu^1^, Dimitrios T. Boumpas^4^, Vana Papaevangelou^1^

##### ^1^Pediatric Rheumatology Unit,3RD Department of Pediatrics, National and Kapodistrian University of Athens, Medical School, Athens, Greece; ^2^2nd Department of Dermatology and Venereology, National and Kapodistrian University of Athens, Medical School, Athens, Greece; ^3^Clinical Immunology-Rheumatology Unit, 2nd Department of Medicine and Laboratory, National and Kapodistrian University of Athens, Medical School, Athens, Greece; ^4^Joint Rheumatology Program,4TH Department of Medicine, National and Kapodistrian University of Athens, Medical School, Athens, Greece

###### **Correspondence:** Evangelia Bountouvi

**Introduction:** Juvenile dermatomyositis (JDM), the most common inflammatory myopathy of childhood, is a rare multisystemic autoimmune vasculopathy, primarily affecting the skin and skeletal muscles.There is mounting evidence that WBMRI can provide invaluable information in the management and following up of JDM patients by evaluating the true extent and inflammatory activity of muscle disease.We report the case of a 5,5 year old girl with JDM and extensive muscle involvement undetected during clinical examination and revealed by WBMRI

**Objectives:** We report the case of a 5,5 year old girl with JDM and extensive muscle involvement undetected during clinical examination and revealed by WBMRI

**Methods:** A 5,5 year old girl presented to our department due to a severe cutaneous eruption and fatigue during the last month. Clinical examination revealed an erythematous, papulosquamous eruption over dorsal surfaces of the knuckles, extensor aspects of the elbows and knees (Gottron’s papules),a malar rash and reduced muscle strength involving mostly the upper body.Muscle strength was assessed using both the Childhood Myositis Assessment Scale (CMAS 38/52) and Manual Muscle Test 8 test (MMT 8 55/80).

**Results:** Laboratory work up revealed a significantly increased creatine kinase, lactate dehydrogenase, aldolase, alanine aminotransferase, aspartate aminotransferase and a moderate increase of inflammatory markers (ESR,CRP).Antinuclear antibodies were positive (1/320) whereas all myositis-specific antibodies (MSAs) tested were negative. WBMRI showed increased signal intensity on T2 and STIR images in almost all muscles of the body and increased signal intensity in the subcutaneous fat tissue of the arms. Intriguingly, WBMRI revealed distal legs and forearm muscle inflammation undetected during clinical examination. Nailfold capillaroscopy demonstrated significant dropout, dilatation and tortuosity of nailfold capillaries. Based on the pathognomonic rash and characteristic findings on WBMRI and nailfold capillaroscopy the diagnosis of JDM was established and invasive diagnostic procedures such as muscle biopsy and EMG were avoided. Aggressive treatment was initiated with both high doses of steroids (pulses of methylprednisolone 30 mg/kg, on 3 consecutive days followed by daily oral prednisolone 2 mg/kg) and high weekly dose of subcutaneous methotrexate. At present, one month after therapy initiation muscle enzymes are within normal range and muscle strength is slightly ameliorated.

**Conclusion:** One of the major challenges in JDM clinical management is to accurately assess disease activity. The potential of musculoskeletal MRI as a non-invasive imaging procedure for defining inflammatory activity of involved muscle is well established and pelvic/thigh MRIs are extensively used. WBMRI screens the entire body with the advantage of evaluating much larger areas of muscles as well as subcutaneous fat tissue, thus providing a complete assessment of total muscular inflammatory burden. This is of utmost importance for the therapeutic optimization in order to prevent further damage Even though WBMRI value has been suggested in small series of JDM patients, its real potential in JDM remains to be explored.

**Disclosure of Interest**

None Declared

## Macrophage activation syndrome

### B9 “Clinical presentation of macrophage activation syndrome in Mexican children with rheumatic diseases”

#### Talia Diaz, Sofia Osorio, Maria Teresa Braña, Yuridiana Ramirez, Luis Aparicio, Andres Rodriguez, Enrique Faugier, Rocio Maldonado

##### Pediatric Rheumatology, Hospital Infantil de Mexico Federico Gomez, Ciudad de Mexico, Mexico

###### **Correspondence:** Talia Diaz

**Introduction:** Macrophage activation syndrome (MAS) is a known complication of pediatric rheumatic disorders, particularly juvenile idiopathic arthritis (JIA), systemic lupus erythematosus (SLE) and Kawasaki disease (KD). MAS is a life-threatening condition with a mortality rate of up to 20%. Therefore, early recognition and immediate therapeutic intervention are critical.

**Objectives:** To describe patient demographics, interventions and outcomes in hospitalized children with MAS complicating SLE, JIA or KD.

**Methods:** Retrospective cohort study of the Children’s Hospital of Mexico Federico Gomez, January 2013 to April 2017. Participants had diagnosis for MAS and either SLE, JIA or KD. The primary outcome was hospital mortality. Secondary outcomes included intensive care unit admission, critical care interventions, and medication use.

**Results:** Seven children met inclusion criteria, including 4 with SLE, 2 with systemic JIA and 1 with Kawasaki disease. Index admission mortality was 28% (2/7). ICU admission (100%), mechanical ventilation (71%), and inotrope/vasopressor therapy (85%) were common. Compared to children with JIA, those with SLE had higher mortality and more ICU care [higher mechanical ventilation duration (10 days vs 2 days, and more time cardiovascular dysfunction]. Children with SLE and JIA received glucocorticoids and cyclosporine at similar rates, but more children with SLE received cyclophosphamide and etoposide. One patient with SLE received etanercept (Anti-TNFa) and another patient with SAM secondary to Kawasaki disease did not need ICU or vasopressor therapy.

**Conclusion:** Organ system dysfunction is common in children with rheumatic diseases complicated by MAS, and children with underlying SLE require more organ system support than children with JIA Current treatment of pediatric MAS varies based on the underlying rheumatic disease. In Mexico, the incidence rate of macrophage activation syndrome secondary to Kawasaki disease is less frequent, the only patient included in this study had a clear clinical and laboratory improvement after treatment with glucocorticoid alone.

**Disclosure of Interest**

None Declared

## Miscellaneous rheumatic diseases

### B10 Erythema nodosum as symptom of systemic diseases

#### Maria Francesca Gicchino, Carmela Granato, Giulia Macchini, Daniela Capalbo, Alma Nunzia Olivieri

##### Department of Women, Child and General and Specialistic Surgery, Università della Campania Luigi Vanvitelli, Naples, Italy

###### **Correspondence:** Maria Francesca Gicchino

**Introduction:** Erythema nodosum (EN)is the most common panniculitis in childhood. The lesions of EN are localized at the lower limbs, in particular in the pretibial region, while upper limbs and trunk are rarely involved. Erythema nodosum can be associated with general symptoms such as fever, weakness, and severe pain, but skin lesions resolve without skin damage.

**Objectives:** To describe five cases of Erythema nodosum in childhood

**Methods:** R.G. f. 3 years old came to our department for a two week history of fever (up to 38 °C) and skin lesions. Physical examination revealed pharyngeal hyperemia and multiple erythematous nodules on the extensor surface of the lower extremities. Laboratory tests showed an elevation of ESR(35 mm/h) and CRP, (1,06 mg/dL) and high levels of Chlamydia Pneumoniae IgM.Throat swab was negative for group A beta-hemolytic Streptococcus (GAS) and her chest X-ray was negative. So antibiotic therapy was prescribed and symptomatology improved.

A.N. m. 8 years old was hospitalized for fever up to 39 °C and skin lesions in the lower limbs. Patient suffered from recurrent abdominal pain and diarrhea. Physical examination revealed abdominal pain and erythematous and painful nodules in pretibial region of both lower limbs. Blood tests showed increase of ESR (40 mm/h) and CRP (1,3 mg/dL). Blood examinations for celiac disease were negative. Fecal calprotectin was high (500 mg/kg). Abdomen ultrasound revealed terminal ileum bowel wall thickening. Crohn’s Disease (CD)was suspected and confirmed with an endoscopy including biopsies.

A.N. f. 5 years old was admitted to our department for fever (38 °C) and skin lesions that started two weeks ago. The patient also had 1 month history of cough. Physical examination revealed: pharyngeal hyperemia, cervical and axillary lymphadenopathy and skin lesions suggestive of erythema nodosum on the extensor parts of both lower limbs. Inflammatory tests were increased (ERS 28 mm/h, CRP 1,5 mg/dL). Her chest X-ray was negative. Mantoux test was positive with an induration of 15 millimeters (mm) after 48 hours and 18 mm after 72 hours, also a Quantiferon test was positive.

L.B. f. 12 years old had an history of fever, headache, fatigue, joint pain and skin lesions.The objective examination revealed: malar rash, arthritis of the right knee, erythematous nodules on the extensor surface of the lower limbs. Blood tests showed anemia (Hb,5 g/dL), thrombocytopenia (PLT 75.000/mm^3^), ERS increased (30 mm/h) positive ANA, antiDNA. Systemic Lupus Erythematous was diagnosed according to ACR criteria

I.L. f. 8 months old had a two months history of recurrent fever and skin lesions. On admission the patient was febrile (TC 38 °C). Physical examination revealed pharyngeal hyperemia, splenomegaly and erythematous nodules on the extensor surface of the lower limbs.Inflammatory tests were increased (ERS 33 mm/h, CRP 2 mg/dL). Antibodies anti CMV, EBV, Chlamydia and Mycoplasma Pneumoniae were negative. Biopsy of a lesion showed a condition compatible with panarteritis nodosa.

**Results:** EN is a skin inflammatory reaction. EN could be associated with infectious diseases (GAS, Chlamydia Pneumoniae, Mycoplasma Pneumoniae, Epstein-Barr virus, Mycobacterium Tuberculosis), drugs, inflammatory bowel diseases, rheumatologic diseases, malignant tumor.

**Conclusion:** The presented cases show that erythema nodosum can be secondary to different diseases.In the diagnostic process associated symptomatology and laboratory tests should be considered to diagnose the disease and to start specific treatment.

**Disclosure of Interest**

None Declared

### B11 Observations from clinical practice

#### Nathan Hasson

##### Paediatric Rheumatology, The Portland Hospital, London, United Kingdom

**Introduction:** The following observations were made from 25 years of practice in Paediatric Rheumatology

**Objectives:** To share these observations to assist others in their practice and to facilitate collaborative research and audit

**Methods:** Review of case data

**Results:** 1. The cause of symptoms in Benign Joint Hypermobility Syndrome relates to children today being physically weak as measured by manual muscle testing (Oxford scale) compared to typical children a generation ago.

2. Chronic Fatigue Syndrome only occurs in children who are hypermobile and weak.

3. Bottom shuffling is a feature of children with hypermobility.

4. W sitting is only seen in hypermobile children as hips can internally rotate 90 degrees and is a safe way to sit

5. Left handedness is only seen in hypermobile children who are clinically right hand dominant but write with their left hand

6. Writing difficulties relate to the fact that grip strength in children is now one third of children a generation ago and not related to pen grip

7. Children sit in a kyphotic posture due to poor core muscle stamina and when sat up straight their left shoulder is higher and narrower than their right due to trapezius muscle spasm

8. Intoeing, outoeing and tip-toe gait often relates to hypermobility and supple subtalar joints.

9. Children with hypermobility in general have higher levels of anxiety than non-hypermobile children. This often leads to symptoms such as abdominal pain.

10. Flat feet are associated with hypermobility and with modern weakness and needs to be corrected with orthotics

**Conclusion:** Hypermobility, which is a normal variant in society, is now associated with a number of problems mostly due to inactivity and weakness in children. These observations should help others in their practice and be the foundation for further research.

**Disclosure of Interest**

None Declared

### B12 First pediatric patient with neuromyelitis optica and Sjogren’s syndrome successfully treated with tocilizumab

#### Achille Marino^1^, Sona Narula^2^, Melissa Lerman^3^

##### ^1^Rheumatology, Meyer Children’s Hospital, Florence, Italy; ^2^Neurology, The Children’s Hospital of Philadelphia, Philadelphia, PA, United States; ^3^Rheumatology, The Children’s Hospital of Philadelphia, Philadelphia, PA, United States

###### **Correspondence:** Achille Marino

**Introduction:** Neuromyelitis optica (NMO) is a central nervous system inflammatory disease characterized most often by optic neuritis (ON) and/or transverse myelitis (TM) along with serum immunoglobulin G antibodies that target the water channel aquaporin-4 (AQP4-IgG). The treatment of this condition is not standardized and may be challenging (1).

**Objectives:** To describe the first child with NMO, and also Sjogren’s Syndrome (SS), successfully treated with the IL-6 receptor humanized monoclonal antibody, tocilizumab.

**Methods:** Case report

**Results:** A 23 month old Caucasian male developed prolonged seizure in the setting of hyponatremia, resolved with hypertonic saline administration. Brain MRI revealed T2 hyperintense lesions involving the brainstem and thalamus. He was diagnosed with central pontine myelinolysis (CPM) and achieved symptomatic remission after treatment with intravenous immunoglobulin (IVIG) and intravenous methylprednisolone (IVMP). Two years later, he developed unilateral acute ON on his right eye, completely resolved following IVMP.

At 11 years, he had an episode of ON in his left eye. There was concern that his presentation could be consistent with NMO, and his serum was positive for anti-AQP4-IgG at that time. Work up revealed positive antinuclear antibody (ANA 1:160) and positive SS-A antibodies, but lip biopsy was inconclusive for SS. Once more, he was treated with IVMP, followed by oral corticosteroids.

At 14 years, the subject developed left-sided vision loss, right hemiparesis, and lethargy. He did not improve with IVMP, but did respond to subsequent PLEX. MRI demonstrated ongoing disease activity with a new large, confluent left-sided supratentorial white matter lesions and confluent T2 hyperintense lesions of the thoracic cord (T2-T5). Given the progression, he was treated with Cyclophosphamide (Cyc) and Rituximab (RTX). After 5 Cyc infusions, he was transitioned to Mycophenolate Mofetil (MMF).

Three months later, he had resolution of spinal involvement on MRI without progression of brain lesions. His MRI incidentally revealed numerous parotid cysts. Given the presence of parotid cysts, positive SSA antibodies and mild symptoms of dry mouth, a diagnosis of SS was made and hydroxychloroquine treatment was started. Over the next two years, he relapsed several times and was treated with IVMP and RTX. He later developed secondary hypogammaglobulinemia and began IVIG replacement. Despite complete CD19^+^ B lymphocyte depletion (absolute CD19^+^ ≤ 2 cells/μl), the relapses occurred with shorter inter-treatment intervals. Therefore tocilizumab was added when he was 16 years old. He was able to discontinue oral corticosteroids within 1 year. Rituximab was stopped after 4 rounds in two years. Over a follow-up period of three years, he has not experienced any relapses, despite absolute CD19+ > 100 cells/μl. He has not developed any adverse effect during ongoing treatment.

**Conclusion:** Blockade of IL-6 may be useful and safe to treat children NMO. RTX is used to treat NMO but it is not always effective. A recent report demonstrated that CD20^−^ plasmablasts (PB), a population not directly impacted by RTX, are increased in the peripheral blood of patients with NMO, and that IL-6 can promote PB survival and anti-AQP4 production in vitro (2). As IL-6 levels are increased in sera and cerebrospinal fluid of patients with active NMO, IL-6 has been hypothesized to be involved in the disease’s immunopathogenesis (3). This may explain why inhibition of the IL-6 pathway can be a valuable option to treat NMO. In this study we describe a pediatric patient who, despite complete depletion of CD19 + B-lymphocytes, did not maintain durable remission with RTX whereas remission on tocilizumab was maintained also after B-cell repopulation.

1. Wingerchuk DM, Banwell B, Bennett JL, et al. International consensus diagnostic criteria for neuromyelitis optica spectrum disorders. Neurology 2015;85:177–189.

2. Chihara N, Aranami T, Sato W, et al. Interleukin 6 signaling promotes anti-aquaporin 4 autoantibody production from plasmablasts in neuromyelitis optica. Proc Natl Acad Sci USA 2011;108:3701–3706.

3. Uzawa A, Mori M, Arai K, et al. Cytokine and chemokine profiles in neuromyelitis optica: significance of interleukin-6. Mult Scler 2010;16:1443–1452.

**Disclosure of Interest**

None Declared

### B13 Pediatric mixed connective tissue disease: variability of clinical characteristics at disease onset in two patients

#### Maria Amelia Muñoz Calonge, Sara Maria Murias Loza, Rosa Maria Alcobendas, Agustin Remesal

##### Pediatric Rheumatology, Pediatrician, Madrid, Spain

###### **Correspondence:** Maria Amelia Muñoz Calonge

**Introduction:** Mixed connective tissue disease (MCTD) is one of the most infrequent rheumatic disorders in children and gathers clinical features of juvenile idiopathic arthritis (JIA), systemic lupus erythematosus (SLE), juvenile dermatomyositis (JDM), and cutaneous systemic scleroderma (CSS). Esophageal dysmotility and cardiopulmonary involvement may occur during the disease course, being the latter which determines morbidity and mortality.

**Objectives:** To describe the main characteristics of two pediatric patients diagnosed with MCTD and substantially different clinical symptoms at disease onset.

**Methods:** Clinical charts review

**Results:** Two patients were included, both girls (case 1 and case 2), whose main characteristics are described in Table 1. Age at disease onset was 9 and 10 years old respectively. Delay of diagnosis ranged between 2 and 3 years. Case 1 was initially diagnosed with positive rheumatoid factor JIA, and case 2 with SLE (being Raynaud phenomenon the main symptom). Inmunology studies were completed by anti-RNP antibodies determination, which were positive in both cases. Both patients met Kasukawa criteria for MCTD. Capillaroscopy showed EMTC pattern in case 1 and sclerodermiform pattern in case 2.

Both patients receive hydroxychloroquine and mycophenolate mofetile.

**Conclusion:** A wide variability exists regarding clinical onset of pediatric MCTD and delay of diagnosis is common. Further research is needed in order to validate paediatric classification/diagnostic criteria.

**Disclosure of Interest**

None Declared

**Table 1 (abstract B13):** Clinical characteristics of two paediatric patients diagnosed with mixed connective tissue disease

	Symptoms at disease onset	Laboratory examinations	Capillaroscopy	Anti-RNP antibodies	Diagnosis
Case 1	Arthritis Arthralgia	ANA 1/2560 ESR 54 mm/h CRP 80.55 mg/dL RF 49.9 UI/mL Lymphopenia	Compatible with MCTD	Positive	MCTD
Case 2	Raynaud’s Disease	ANA 1/2560 Positive Anti-Ro antibodies C4 9 mg/dl ESR 28 mm/h Lymphopenia	Sclerodermiform-like	Positive	MCTD

*ANA* antinuclear antibodies, *ESR* erythrocyte sedimentation rate, *CRP* C-reactive protein, *RF* rheumatoid factor, *MCTD* mixed connective tissue disease

### B14 Complex regional pain syndrome type I: therapeutic management and progosis factors

#### Esmeralda Núñez-Cuadros^1^, Rocio Galindo Zavala^1^, Gisela Díaz-Cordovés Rego^1^, Cristina Antúnez Fernández^2^, Yaiza García Molina^2^, Antonio L. Urda Cardona^2^

##### ^1^Paediatric Rheumatology, Universitary Regional Malaga Hospital, MALAGA, Spain; ^2^Paediatrics, Universitary Regional Malaga Hospital, MALAGA, Spain

###### **Correspondence:** Esmeralda Núñez-Cuadros

**Introduction:** Complex regional pain syndrome (CRPS) is a neuropathic disoder characterized by chronic pain of unknown cause that associates sensorial, autonomic and motor symptoms. It is an underdiagnosed entity that requieres a multidisciplinary approach. Currently there are no standarized treatment protocols.

**Objectives:** To describe clinical and epidemiological features of CRPS patients and to analize factors potentially involved in evolution and therapeutic response.

**Methods:** Descriptive retrospective study in CRPS patiens (diagnosed accoding to Budapest criteria) younger than 14 years monitored by a Pediatric Rheumatology Unit between January 2009 and February 2017.

**Results:** 15 patients were enrolled, 11 women mean age 10,82 years (±1,7 SD). Among possible triggers we found previous trauma in 12 patients (11 were inmovilized with a median duration of 20 days) and we identified unfavorable psychological factors in 7 patients. All of them showed unilateral lower limbs involvement, except for one patient with bilateral affection.

In terms of clinical symptoms, 14 presented functional impairement, 13 neuropathic pain (associated to allodynia in 10) and 5 trophic changes. None of them had hypertrichosis. The mean time of diagnosis delay was 55,1 days (±63,2 SD). Magnetic resonance was performed in 14 patients and showed pathologic findings in 9. Bone scintigraphy was performed in 12 patients, being diagnostic (hypercaption in early phase) in 8.

Before the diagnosis was done all the patients received NSAIDs without improvement. After the diagnosis everyone began physiotherapy and pharmacologic treatment: pregabaline in 12, minor opioids in 8 and topical capsaicine 8% in 4. Due to refractoriness, a bupivacaina epidural catheter was used in 3 patients.

Currently, 9 patients has no symptoms, with a median time of resolution of 267,8 days (IQR:135–330) and 6 patients are still being monitored because of persistent symptoms, with a median time of evolution of 246 days (IQR: 94–342).

At the bivariant analysis no relation was found between time until resolution and duration of immobilization neither unfavorable psychological factors, but we found a strong possitive correlation with the time of diagnostic delay (Rho: 0,85; p = 0,007).

**Conclusion:** Frequently there is a trauma history in CRPS in children but it is not always present.

Althoug imaging tests are really helpfull to establish the diagnosis, sometimes they do not show any changes.

It requires a multidisciplinary approach with physiotherapy and analgesia as the fundamental basis of it.

The presence of desfavourable psycological factors in nearly a half of patients makes it essential a professionalized aproach to this level to manage an adequate control of the disease.

An early diagnose determines a faster clinical resolution, that is why a high suspicion rate is needed.

**Disclosure of Interest**

None Declared

### B15 A rare cause of joint pain: synovial haemangioma

#### Nihal Sahin^1^, Habibe S. Durmus^2^, Ayse S. Pinarbasi^3^, Zubeyde Gunduz^1^, Muammer H. Poyrazoglu^1^, Zehra F. Karaman^4^, Turhan Oktem^5^, Mithat Oner^6^, Ruhan Dusunsel^1^

##### ^1^Pediatric Rheumatology, Erciyes University Faculty of Medicine, Kayseri, Turkey; ^2^Pediatrics, Erciyes University Faculty of Medicine, Kayseri, Turkey; ^3^Pediatric Nephrology, Erciyes University Faculty of Medicine, Kayseri, Turkey; ^4^Radiology, Erciyes University Faculty of Medicine, Kayseri, Turkey; ^5^Pathology, Erciyes University Faculty of Medicine, Kayseri, Turkey; ^6^Ortopedics, Erciyes University Faculty of Medicine, Kayseri, Turkey

###### **Correspondence:** Nihal Sahin

**Introduction:** Synovial haemangioma is a rare type of tumor which affects the knee joint. Patients who are usually female and their second decade of life, often apply nonspesific symptoms like pain and limitation of motion. When this uncommon tumor is untreated, it can cause cartilage erosion and degenerative joint disease. Because of its rarity, physician awareness is low that results in delayed diagnosis. We presented a synovial haemangioma of the knee to increase awareness of pediatricians.

**Objectives:** Clinical case

**Methods:** A 17-year-old girl was admitted to Erciyes Unversity Faculty Of Medicine Peidatric Rheumatology in January 2017 with complaints of pain, swelling and tenderness in her left knee.

**Results:** A 17-year-old girl was admitted with complaints of pain, swelling and tenderness in her left knee since she was 3 years old. With the previously magnetic resonance imaging of the knee joint, she was diagnosed as chronic arthritis on the knee joint. Non-steroidal anti-inflammatory drugs were recommended. Patient applied to our hospital because of ongoing complaints. There was no any arthritis finding including tenderness, pain on motion, deformity, and limitation of motion. On the upper part of left knee, she had localized swelling measuring 3,5x4 cm in diameter. Magnetic resonance imaging (MRI) showed synovial haemangioma characterized by a well-circumscribed contour with a lobule filling the left suprapatellar bursa and space- filling formation with heterogeneous intense contrast enhancement after contrast agent administration. It was required open total synovectomy and mass resection, and histopathological findings were compatible with cavernous haemangioma.

**Conclusion:** Tumor and tumor like lesions such as synovial haemangiomas should be kept in mind in patients with arthritis- compatible findings in single joint. In case of doubt, especially intra-articular lesions, imaging methods should be used for early diagnosis and prevent long term sequel.

**Disclosure of Interest**

None Declared

### B16 Bowel bypass syndrome after postnatal jejunoileal anastomosis

#### Gordana Susic^1^, Tamara Krstajic^2^, Dragana Vujovic^2^, Nedeljko Radlovic^2,3^, Zoran Lekovic^2,3^, Dusica Novakovic^1^, Gordana Milosevski Lomic^2^

##### ^1^Institute of Rheumatology, Belgrade, Serbia; ^2^University Children’s hospital, Belgrade, Serbia; ^3^School of Medicine, University of Belgrade, Belgrade, Serbia

###### **Correspondence:** Gordana Susic

**Introduction:** Rheumatic manifestations are not uncommon complication of jejunoileal bypass in treating morbid obesity, but also in other conditions without reduction of lenght small intestini. Pathogenesis is poorly understood, but overgrowth of intestinal bacteria may play causative role.

**Objectives:** To present unusual case of reactive arthritis

**Methods:** data from the medical history

**Results:** We are presenting 18 months old girl at the first admission in our clinic, with signs of arthritis appeared 14 months after bowel resection due to prenatal volvulus. She was underwent to the surgical procedure in the first day after birth, and jejunoileal anastomosis was done, leaving only 9 cm small intestine, ileocoecal valve was spared. Febrile episode with symptoms of mild respiratory infection proceeded to rheumatologic manifestation a few days before that. Personal history: weight at the birth 3400 g, Apgar score 8/9, delivery on time, not vaccinated, total parenteral nutrition for 7 months, after that time special diet was conducted. Clinical examination showed malnourished girl (8350 g below P3), not walked alone, otherwise healthy, with evident polyarthritis: left wrist, MCP and PIP on the left hand, both knees, left ankle, dactylitis of II finger of the left foot and limited range of motion in left MTP area. Arthritis was confirmed by ultrasound examination. There were no any skin changes, the belly was meteoristic, but liver and spleen were within normal range.

Results of the laboratory tests showed: CRP 73,6 mg, ESR 16 mm/h, WBC 12,4 x 10^9^/L, Hb 130 g/l, PLT 324 x 10^9^/L, IgM 3,02 g/L and IgG 17,5 (above upper limit) with elevation of all subclass of IgG, HLA -A1, 32, B35.

Results of the following laboratory studies were normal: blood cultures, culture throat and urine, for hepatitis B surface antigen, antibody to hepatitis A virus, rheumatoid factor, C4, C3, total complement, ANA, ANCA, C3, C4, CIC, IgA. Several samples of urine revealed hematuria. Broad-spectrum antibiotic – Cephtriaxone was introduced, later after consultation with nephrologist was changed with sulfamethoxazole/trimetoprine 5 mg/BW, which she continued to take at home. Probiotic and loperamide were standard medication after surgical procedure, and ibuprofen was added due to arthritis. On the day of discharge from hospital, after 15 days from admisison, the girl was in good condition, with normal laboratory analysis, without any signs of arthritis. Hematuria was explaned by hypercalciuria due to malabsorptional syndrome, and potassium citrate was introduced for prevetion of hypercalciuria. Several motnhs later rickets was confirmed clinicaly and by x-ray, and vitamin D suplementation was added. Up to now the girl had several relapses of arthritis, with limited duration, resolving of symptoms for a few days. She has normal renal function, temporary hipercalciuria, mild low mass wieght proteinuria and propensity to hiperoxaluria. Repeated diarrhea is the main problem sometimes leading to metabolic acidosis and requiring antibiotic treatment.

**Conclusion:** We report the patient with unusual type of reactive arthritis. To our best knowledge this is the youngest patent with bypass syndrome so far published. Prophylactic treatment with antibiotic has been showed good results in suppressing joints inflammation, however this is self-limiting condition. Other manifestations of malabsorption require serious approach and treatment.

We thank to the parents for cooperation. A written consent was obtaned for the publication of the case report.

**Disclosure of Interest**

None Declared

## Psycho-social aspects and rehabilitation

### B17 Nurse’s experiences of distraction during procedures in pediatric care

#### Karina Mördrup^1^, Gunilla Hesselstrand^2^

##### ^1^Pediatric Rheumatology Unit, Karolinska University Hospital, Stockholm, Sweden; ^2^Pediatric Orthopedic Unit, Karolinska University Hospital, Stockholm, Sweden

###### **Correspondence:** Karina Mördrup

**Introduction:** Children’s experiences of fear and pain from hospital visits could result in subsequent visits feeling even more daunting and negative experiences could follow the child into adulthood. Earlier studies shows that preparing and distraction in a playful manor could decreased the child’s experience of pain and fear during a painful procedure. Parents and children should be involved in distraction and communication about the child’s care. The nurse has, together with the child, responsibility to select the distraction method and show how to use it.

**Objectives:** To describe nurses’ experiences of distraction associated with painful and unpleasant procedures in pediatric care.

**Methods:** Descriptive study design using qualitative interviews with six nurses from pediatric care were conducted. To process the data qualitative content analysis was used.

**Results:** The result could be divided into two categories; individualized care and interaction. The result were clarified of nine subcategories.

**Conclusion:** The nurse’s used distraction in almost all the painful procedures that children went through. Individualized distraction, safety, allocating time and participation of the child was crucial for effective distraction. Honest communication and sensitivity to the child’s experiences created the conditions for a good interaction. Cooperation with parents and colleagues helped the distraction process for child and nurse. Evaluation of the distraction was made by assessing the child’s body language.

**Disclosure of Interest**

None Declared

## Spondyloarthritis (SpA) and enthesitis related arthritis (ERA)

### B18 Simultaneous presentation of musculoskeletal and gastrointestinal symptoms in HLA-B27 positive and anti-TTG negative patient with typical celiac disease histology

#### Iva Sorić^1^, Lovro Lamot^1,2^, Mandica Vidovic^1^, Mirta Lamot^1^, Miroslav Harjacek^1,2^

##### ^1^Clinical Hospital Center Sestre Milosrdnice, Zagreb, Croatia; ^2^Univeristy of Zagreb School of Medicine, Zagreb, Croatia

###### **Correspondence:** Iva Sorić

**Introduction:** in recent years a number of studies showed a connection between intestinal dysbiosis and various immunological diseases, hence it is not coincidence gastrointestinal abnormalities can often be found in patients with rheumatic diseases and musculoskeletal symptoms are not rare in variety of digestive system disorders. Nevertheless, when both musculoskeletal and gastrointestinal symptoms presents simultaneously at the beginning of the disease, it might be difficult to distinguish the primary disorder.

**Objectives:** to present a patient with simultaneous presentation of characteristic gastrointestinal and musculoskeletal symptoms.

**Methods:** case report.

**Results:** a thirteen year old boy with medical history of infantile colic and a brother with HLA B27 positive juvenile spondyloarthritis was admitted to our department due to persistent inflammatory lumbosacral pain, Achiles enthesitis, and abdominal discomfort. During the six months period prior to the admission he had six episodes of fever, cough, abdominal pain and aphthae lasting for few days. Acute surgical disease was excluded on several occasions. Chest radiograph, throat and nose cultures showed no signs of infection. During the last few episodes he developed swelling of the right talocrural joint which resolved after short course of NSAID. Later, severe pain in sacroiliac (SI) joints with morning stiffness emerged, while his stools became loose and frothy. Abdominal ultrasound showed ileocecal mesenteric lymphadenitis and dilated intestinal loops filled with dense liquid content. Upon the first examination by paediatric rheumatologist he had palpatory periumbilical pain as well as tenderness of SI joints and Achiles entheses. His height and weight were below the 5^th^ centile while estimated bone age was 12. ANA, ANCA and RF were negative. There was no sign of uveitis on ophthalmologic examination. HLA typing was positive for B27. Radiographic and magnetic resonance imaging of SI joints and spine showed no signs of inflammation. Immunoglobulin A anti-tissue transglutaminase antibody (IgA TTG) was negative while serum IgA levels were normal. No pathological agents were isolated from stool. Several faecal occult blood tests were negative and faecal calprotectin levels were normal. Endoscopy of upper and lower gastrointestinal tract revealed hiatal hernia, oesophagitis, gastritis and duodenitis. Histologic evaluation of several duodenal biopsy specimens displayed typical features of celiac disease. A month after initiation of gluten free diet and NSAID’s the improvement of all symptoms was noticed.

**Conclusion:** although they differ in HLA predispositions, both JIA and CD are associated with 4q27 locus, which encodes IL-2 and IL-12 cytokines important for activation and regulation of immune system, and both are linked with a functional single nucleotide polymorphism in the PTPN22 gene (1858C > T). Intriguingly, lymphocyte cytotoxicity in the intestinal mucosa is abnormally increased in both entities, suggesting some luminal, possibly a nutritional factor, may be involved. This agent is well established in CD, but number of cases, including presented, exhibited beneficial role of gluten free diet in arthritis patients as well. Our patient clearly met ILAR criteria for ErA and had typical histological features of CD, but without elevated anti-TTG levels. Since his musculoskeletal symptoms could also be explained as a part of extra-intestinal CD manifestations, the real distinction of underlying disorders was challenging. Therefore it is reasonable to conclude both CD and certain subtypes of JIA have multiple shared mechanisms making them hard to separate in given circumstances.

**Disclosure of Interest**

None Declared

## Systemic juvenile idiopathic arthritis (JIA)

### B19 Reaching the limits despite extended immunomodulatory therapy in a patient with systemic JIA; a case report

#### Eva Adank, Elvira Cannizzaro Schneider

##### Department of Paediatric Rheumatology, Kinderspital Zürich, Zürich, Switzerland

###### **Correspondence:** Eva Adank

**Introduction:** New immunomodulatory therapies that have emerged over the past years have greatly improved success in autoimmune disease treatment. We present a case of refractory systemic JIA (sJIA) that despite extended medical treatment shows activity of the disease.

**Objectives:** As this case with exhausted use of new immunomodulatory drugs illustrates the limitation of therapeutic options available for sJIA it shows on the other side a good safety profile without severe side effects.

**Methods:** Case Report

**Results:** An eleven year old girl presented with typical signs of sJIA with recurring fever over 2 weeks, typical rash, and splenomegaly. After stopping the oral steroids reactivation of the disease with gonarthritis and fever occurred both under the treatment with NSAID (Naproxen) and Methotrexate (MTX) injections (17.5 mg/week). Even the intravenous therapy with Tocilizumab (8 mg/kg every 2 weeks) and steroids didn’t show any sufficient effect as fever, rash and arthritis persisted. A first attempt with Canakinumab (150 mg) failed because of high fever and arthritis 10 days after the first injection, which was continuing after the premature second injection after 24 days already. As a next step we installed Anakinra with an initial dose of 40 mg/day (1 mg/kg/day).The dose needed to be raised because of signs of systemic inflammation and after an intravenous steroid pulse treatment we reached temporary stability at the dosage of 100 mg daily (2.4 mg/kg/day) still under high dosage of steroids (60 mg daily). An exacerbation presenting as MAS with rapidly raise of ferritin to 5850ug/l along with other activity values caused an additional therapy with Cyclosporine and the elevation of Anakinra to 200 mg daily (4.7 mg/kg/d) after treatment of MAS with intravenous steroid pulse therapy. Under this treatment a stabilization of her condition was seen including disappearance of fever and normalization of the laboratory values. Gradually we could taper the oral steroids over 8 month from 60 mg daily to 10 mg daily, but under the dosage of 10 mg (0.23 mg/kg/d) signs of systemic inflammation returned. As an immunomodulatory effect as well as to prevent septic complications due to long lasting treatment with steroids we started intravenous immunoglobulin (0.4 g/kg) monthly and Posaconazol as antifungal prophylaxis. While again rising the steroid doses up to 60 mg/day and briefly to 80 mg/d our patient still presented with intermitting fever and high activity signs in the lab. This brought us to change again the IL-1-antagonist to Canakinumab after about one year of therapy with Anakinra that in fact also caused strong local reaction due to the injections that were applied twice daily. During 11 month of therapy with Canakinumab, gradually raised from 300 mg/3 weeks to 300 mg/2 weeks, we had at least for a short time a satisfactory response with low lab activity and no fever under 60 mg/d Prednisolon and 300 mg/2 weeks Canakinumab. But the attempt to reduce the steroid therapy failed again. At a dose of oral steroids of 50 mg/day persisting fever with signs of inflammation in the lab occurred.

At least in contrast to prior therapeutic setting with this extended treatment (fourfold dose of Canakinumab, high-dose of oral steroids, intravenous immunoglobulin monthly, additionally Cyclosporine and MTX) the only clinical symptom was fever. Some lab values (LDH, AST, ALT, CRP, ESR) showed little activity, but no rush, no arthritis and no signs of MAS were seen as a partial respond to this treatment. For all that high immunosuppressive therapy we did not have any severe infections nor other serious side effects beside short stature and the cataract due to the long lasting steroid therapy.

**Conclusion:** This exemplary case illustrates the still ongoing three-year search for effective therapy in a patient with sJIA, the whole spectrum of current therapeutic possibilities and its safety profile concerning severe infections. It also shows us that even with new immunomodulatory therapies there are still limitations in controlling the disease.

**Disclosure of Interest**

None Declared

## Systemic lupus erythematosus and antiphospholipid syndrome

### B20 Growth in children with Juvenile Systemic Lupus erythematosus in the Sultanate of Oman

#### Eiman Abdalla^1^, Irfan Ullah^1^, L Jeyaseelan^2^, Reem Abdwani^1^

##### ^1^Child health, Sultan Qaboos University Hospital, Muscat, Oman; ^2^Biostatistics, Sultan Qaboos University Hospital, Muscat, Oman

###### **Correspondence:** Eiman Abdalla

**Introduction:** Systemic lupus erythematosus (SLE) is a chronic, multisystem autoimmune disease with juvenile onset SLE representing 10-20% of all SLE cases and with earlier diagnosis and better approaches to treatment, there has been marked improvement in the prognosis but with more morbidities seen, such as growth failure and pubertal delay.

**Objectives:** The goal of our study is to assess growth of Omani children with juvenile onset SLE, by evaluating their anthropometric measurements, identifying growth failure and study contributing factors to growth failure.

**Methods:** This is a retrospective study in which the serial anthropometric measurements (weights and heights) of patients with juvenile onset systemic lupus erythematosus (jSLE) following in pediatric rheumatology, child health department in SQUH are retrieved from their records. Inclusion criteria include children below 16 yrs. of age who are diagnosed with jSLE as per 1982 revised ACR criteria. Anthropometric measurements are obtained at disease onset, 6 months, 12 months and 24 months after disease onset. Parents’ heights are measured to calculate the child’s respective sex-adjusted mid parental height (target height). This is calculated by adding 2.5 inches or 6.5 cm to the mean of the parents’ heights for boys while for girls, subtract 2.5 inches or 6.5 cm from the mean of the parents’ heights. Parent-adjusted height z score is calculated as the difference between height z score for chronological age and target height. Growth failure was defined as parent-adjusted height z score less than −1.5. Height deflection (decreased height z score > − 0.25 per year) compared to baseline was evaluated. Factors which might have attributed to growth failure were studied and these included growth failure at baseline, disease duration, disease activity (SLEDAI score) and cumulative steroid dose.

**Results:** Twenty five out of 31 patients have complete data (6 patients could not get either of parents height as only one parent come with patient, 2 patients are no longer following with us). Growth failure is identified in 8 patients (female: male ratio of 7:1) hence the hospital prevalence and the 95% CI of growth failure is 32% (13.7%, 50.2%) in our study. The female prevalence of growth failure is 35% as compared to male prevalence 20%. However, the difference was not statistically significant (p value 0.915). Growth failure determinants were previous growth failure at initial assessment (p 0.00) and high cumulative corticosteroid doses (p 0.061), while disease duration and disease activity were not found to be statistically significant contributing factors (p value 0.24 and 0.52, respectively).

**Conclusion:** Growth failure was identified in our children with juvenile SLE, and those at risk were those who already had growth failure previously and those who received high corticosteroid dose.

**Disclosure of Interest**

None Declared

### B21 Maternal and neonatal outcome of SLE pregnancies in Sultan Qaboos University Hospital

#### Reem Abdwani^1^, Laila AL Shaqsi^2^, Ibrahim Al Zakwani^1^

##### ^1^Child Health, Sultan Qaboos University, Muscat, Oman; ^2^Child Health, Oman Medical Speciality Board, Muscat, Oman

###### **Correspondence:** Reem Abdwani

**Introduction:** SLE s an autoimmune disease that affect women primarily of childbearing age. As a consequence, pregnancy and its neonatal outcome are of particular importance in this condition. It appears that individual races and ethnicities may exhibit differences in disease susceptibility and disease manifestations. There are limited studies in SLE patients emanating from Arab world, however, it is recognized that Arabs experience a different disease burden than Caucasians

**Objectives:** To determine differences in maternal and neonatal outcomes in pregnancies complicated with systemic lupus erythematosus (SLE) compared to those with normal pregnancies in Arab women from Sultanate of Oman.

**Methods:** A retrospective analysis of 147 pregnant mothers with their corresponding infants was enlisted (56 (38%) SLE mothers and 91 (62%) pregnant controls). Analyses were performed using descriptive statistics.

**Results:** The overall mean age of the cohort was 30 ± 5 years ranging from 19 to 44 years. The SLE mothers were mainly treated with hydroxychloroquin (n = 41; 73%), prednisolone (n = 38; 68%) and azathioprine (n = 17; 30%). Disease activity revealed that 39% (n = 22) had no disease activity while 41% (n = 23), 13% (n = 7) and 7.1% (n = 4) had mild (SLEDAI, 1–5), moderate (SLEDAI, 6–10) and severe (SLEDAI, ^3^11), respectively. The SLE mothers were associated more with abortions (43% *vs.* 15%; *p* < 0.001), gestational diabetes (28% *vs.* 10%; *p* = 0.004), polyhydramnious (7.1% *vs.* 0; *p* = 0.020), pre-term labor (21% *vs.* 1.1%; *p* < 0.001), previous pre-term (8.9% *vs.* 1.1%; *p* = 0.030) and intra-uterine growth retardation (21% *vs.* 0; *p* < 0.001) when compared to normal pregnant controls. Furthermore, the neonates born to SLE mothers were more likely to be pre-term (29% *vs.* 1.1%; *p* < 0.001), had low birth weight (<2500 g) (32% *vs.* 1.1%; *p* < 0.001) and associated with still birth (7.1% *vs.* 0; *p* = 0.010) when compared to neonates born to normal pregnancies (Table 2).

**Conclusion:** SLE mothers were associated with worse neonatal and maternal outcomes when compared to normal pregnant mothers despite adequate disease control and antenatal follow up.

**Disclosure of Interest**

None Declared

**Table 2 (abstract B21):** Demographics of Maternal Pregnancies

Characteristic, *n (%) unless specified otherwise*	All (N = 147)	Non-SLE (n = 91)	SLE (n = 56)	*P*-value
Age, mean ± SD, years	30 ± 5	29 ± 5	31 ± 5	0.145
Primigravida (1)	35 (24%)	24 (26%)	11 (20%)	
Multigravida (2–4)	79 (54%)	52 (57%)	27 (48%)	0.084
Grand multigravida (>5)	33 (24%)	15 (16%)	18 (32%)	
Abortions	38 (26%)	14 (15%)	24 (43%)	<0.001
Gestational diabetes	24 (17%)	9 (10%)	15 (28%)	0.004
Polyhydramnios	4 (2.7%)	0	4 (7.1%)	0.020
Pre-term labour	13 (8.8%)	1 (1.1%)	12 (21%)	<0.001
IUGR	12 (8.2%)	0	12 (21%)	<0.001

### B22 Juvenile Systemic Lupus Erythematosus (J SLE) initially diagnosed as autoimmune hepatitis (AIH) -case report

#### Erato Atsali^1^, Pelagia Katsimbri^2^, Antonis Fanouriakis^2^, Sorina Boiu^1^, Vana Papaevangelou^1^, Dimitrios T. Boumpas^2^

##### ^1^Pediatric Rheumatology Unit,3RD Department of Pediatrics, National and Kapodestrian University of Athens, Medical School, Athens, Greece; ^2^Joint Rheumatology Program, 4TH Department of Medicine, National and Kapodestrian University of Athens, Medical School, Athens, Greece

###### **Correspondence:** Erato Atsali

**Introduction:** Hepatic involvement in patients with SLE is well documented, but considered to be rare. To distinguish whether elevated liver enzymes in patients with SLE represent a non-specific hepatic involvement or AIH remains a difficult challenge for the treating physician.

**Objectives:** We present the case of a 17,5 years old girl with Juvenile Systemic Lupus Erythematosus initially diagnosed as autoimmune hepatitis.

**Methods:** A 15,5 years old girl with the diagnosis of autoimmune hepatitis presented to our department two years ago seeking a second opinion. Laboratory exams done due to a persistent maculopapular rash of hands, elbows and feets (considered initially as contact dermatitis) had revealed a significant increase of transaminase levels (4fold above the upper normal limit). Gamma glutamyl transpeptidase, rheumatoid factor IgM and immunoglobulin G were also elevated. A more extensive laboratory work up excluded viral hepatitis A,B and C, Wilson disease and celiac disease. Serological work up revealed positive antinuclear (ANA), smooth muscles antibodies (SMA) and anti liver cytosol type 1 (anti –LC-1) antibodies.IgM antibodies against Coxsackie virus, ECHO virus and Epstein Barr virus were also positive. Liver biopsy was performed and revealed no pathology. Based on serological markers the diagnosis of AIH was considered as most probable and the girl was started on per os prednisolone therapy (0,5 mg/kg).

**Results:** On physical examination the girl had reticular peliosis, arthritis of metatarsophalangeal joints and a papular rash of hands, elbows and feet with no clinical sign of chronic liver disease (jaundice,firm liver,hepato/spleno megaly,palmar erythema).Based on normal liver histology we considered the diagnosis of autoimmune hepatitis as less probable. Supplemental laboratory exams revealed low complement C3 and positive anti Ro antibodies and Coombs test.Cardiological examination showed a small pericardial effusion. SLE was diagnosed and hydroxychloquine was added to treatment. Nine months later,while receiving a low dose of steroids,she developed a typical malar rash and nephritis with significant proteinuria and hypoalbuminemia. A renal biopsy showed early membranous nephritis. Treatment with pulses of methylprednisolone (3 pulses on 3 consecutive days,1gr each), followed by per os steroids and azathioprine were started leading to a complete resolution of malar rash, cutaneous eruptions and proteinuria.Steroids were slowly tapered. The girl is now treated with hydroxychloquine,azathioprine (2,5 mg/kg) and a low dose of prednisolone (5 mg/day).SLE activity remains low (SLEDAI 2 K:2).

**Conclusion:** Specific markers for AIH are soluble liver antigen (SLA), liver-pancreas, smooth-muscle antibody (SMA) with specificity for F-actin and microsomal autoantigens, such as anti-liver kidney antibodies (anti-LKM antibody). While these markers may help to differentiate AIH from SLE serologically, liver histopathology represents the key feature that distinguishes AIH in SLE from nonspecific hepatic involvement in SLE. In patients with AIH liver histopathology shows characteristic lesions, such as interface hepatitis, rosetting of hepatocytes, emperipolesis and - consecutive to inflammation - fibrosis. As the diagnosis of AIH in SLE patients can influence treatment choices, longterm outcome and optimal surveillance of the patients, adequate attention should be taken to differentiate between “true additional” AIH and secondary liver involvement.

**Disclosure of Interest**

None Declared

### B23 Pyrexia of Unkown Origin (PUO) and severe ventricular systolic dysfunction – presenting manifestations of childhood SLE in a 15 year old boy

#### Mahesh Janarthanan^1^, Dhanarathnamoorthy Vetrichelvan^2^, P Ramachandran^2^, Sangeetha Geminiganesan^2^, Dinesh Kumar^2^, Subba Rao^2^

##### ^1^Division of Pediatric Reumatology, Sri Ramachandra University, Chennai, India; ^2^Department of Pediatrics, Sri Ramachandra University, Chennai, India

###### **Correspondence:** Mahesh Janarthanan

**Introduction:** Cardiac manifestations in childhood SLE are not uncommon. Up to 50% of patients can have cardiac manifestations including pericarditis, pericardial effusion or valvular vegetations.A significant proportion of these patients do not have cardiac related symptoms and these manifestations are picked up on screening by echocardiogram.

**Objectives:** Severe ventricular systolic dysfunction is very rare as a presenting manifestation in childhood SLE.

**Methods:** We present the case of a 15 year old boy with childhood SLE who presented to us with pyrexia of unknown origin and severe ventricular systolic dysfunction needing immune modulators,inotropic and ventilator support.

**Results:** A 15 year old boy presented with history of fever for 15 days and 2 episodes of generalized tonic,clonic seizures. He did not have any other features suggestive of connective tissue disorder.His investigations on admission revealed Hb-10.9gms/dl, Total white cell count- 5400cells/cumm, Polymorphs 79%, Lymphocytes −15.9%, Platelet count 122000,ESR – 71 mm/hr. Direct Coomb’s test was negative.His liver function tests showed transaminitis SGOT 442U/l and SGPT 131 U/l. Serum uric acid 8.6 mg/dl and serum LDH 2387 U/l were elevated. Serum creatinine was 1 mg/dl and urine routine showed 1+ proteinuria with few red blood cells. Bone marrow examination revealed reactive changes.

Cerebro spinal fluid analysis and MRI brain were normal. He was commenced on empirical antibiotics and antiviral agents. On day 3 of admission to hospital he developed breathing difficulty with dropping of blood pressure. Chest Xray showed cardiomegaly.Echocardiogram at this stage revealed severe left ventricular dysfunction with an ejection fraction of 20% and mild pericardial effusion. He was treated with ventilatory and inotropic support.The results of his autoimmune work were now available and this revealed ANA positive 1:320 and low complement levels. dsDNA, ENA and APLA work up were negative.

He was treated with dobutamine, milrinone,spirinolactone and furosemide infusion. He also received pulsed methylprednsiolone, IVIG and cyclophosphamide.The patients hemodynamic status improved gradually and he was weaned off inotropic and ventilatory support over 2 weeks. After he stabilized a renal biopsy was done and this revealed class III lupus nephritis. The patient is currently on a maintainence regimen with low dose steroids, hydroxychloroquine, mycophenolate mofetil and enalapril.

**Conclusion:** Severe ventricular dysfunction with very low ejection fraction as presenting cardiac manifestation of SLE has rarely been reported in childhood SLE. Combination of supportive measures, pulsed high dose methylprednisolone, IVIG and cyclophosphamide was life saving in this patient.

**Disclosure of Interest**

None Declared

### B24 Systemic Lupus Erythematosus in a female with X trisomy

#### Eleni-Maria Papatesta, Despoina Maritsi, Irini Eleftheriou, Maria Tsolia, Olga Vougiouka

##### Second department of Pediatrics, P. & A. Kyriakou Children’s Hospital, Athens, Greece

###### **Correspondence:** Eleni-Maria Papatesta

**Introduction:** Systemic Lupus Erythematosus (SLE) is the prototype of autoimmune diseases which affects mostly women of childbearing age. It is estimated that for 15-20% of all patients, the onset of the disease is in childhood. Recent evidence suggests that genetic factors transmitted by the X-chromosome may confer increased risk for SLE and autoimmunity in general. The calculated prevalence of SLE in females with triple X syndrome (47, XXX), which accounts for 1 in 1000 births, is three times higher than in 46XX females. It is also known that SLE is more common in males with Kleinfelter syndrome (47, XXY) rather than in genotypic 46XY.

**Objectives:** We present a patient with mosaic 47ΧΧΧ (90%) and 46ΧΧ (10%) who was diagnosed with SLE

**Methods:** Case report

**Results:** A 13-year old girl presented to our hospital with pain and swelling of her right hand and her left foot, asthenia, headaches, mouth sores and a rash on her legs. Symptoms initiated 3 weeks prior to admission. Prenatal screening had shown a mosaic embryo with two cell lines 47, XXX (90%) and 46, XX (10%).

On examination she had arthritis of her right wrist and left ankle joints, a diffuse purpuric rash primarily affecting her lower limbs, facial erythema and mouth ulcers. Examination of her cardiovascular, respiratory, gastrointestinal and central nervous systems was intact. There was no lymphadenopathy. On admission, laboratory tests revealed marginal leucopenia (WBC 4000(N 50.8%/L 41.1%/M 6%), Coombs positive hemolytic anemia, (Hb 9.7 g/dl) and an ESR of 40 mm/h. Renal function tests showed urea of 53 mg/dl (nv 5–44) and creatinine 0,9 mg/dl (nv 0.4-0.8). Moreover, antinuclear antibody titer >1/640, diffuse, positive mitotic pattern, anti-dsDNA positive (RIA >90), low complement levels C3 43.8 mg/dl C4 4.26, CH50 < 10, anti ENA positive, anti SSA (Ro) positive. Urinanalysis showed severe proteinuria and hematuria, in keeping with active nephritis. The result of the renal biopsy was diffuse proliferative glomerulonephritis with individual membranoproliferative ‘standard’, findings consistent with SLE nephritis IV-G class (A). During her hospitalization her vital signs remained stable.

Her medical management included methylprednisolone pulses followed by slow corticosteroid tapering, mycophenolate mofetil and hydroxychloroquine.She adequately responded to treatment and is on a regular follow up.

**Conclusion:** For years SLE female predilection was considered as a hormonal hypothesis. Recent data on epigenetic X-chromosome non deletion on somatic female cells support the phenomenon of gene overexpression on X-chromosomes as responsible for female predisposition. X-polysomy with its abundance of X-chromosomes may represent a nature’s experiment on the effect of gene dosage on the expression and pathogenesis of SLE. Thus, X-polysomy should highly suspected for SLE.

**Disclosure of Interest**

None Declared

## Treatment

### B25 Canakinumab experience from a pediatric rheumatology center in Istanbul

#### Mustafa Çakan^1^, Nuray Aktay Ayaz^1^, Şerife Gül Karadağ^1^, Gonca Keskindemirci^2^

##### ^1^Pediatric Rheumatology, Kanuni Sultan Süleyman Research And Training Hospital, İstanbul, Turkey; ^2^Pediatrics, Kanuni Sultan Süleyman Research And Training Hospital, İstanbul, Turkey

###### **Correspondence:** Nuray Aktay Ayaz

**Introduction:** Canakinumab is a human monoclonal anti-IL-1β antibody that neutralizes the activity of IL-1β by binding to it. Canakinumab is used effectively in many of the AIDs.

**Objectives:** The aim of this study is to demonstrate diagnosis, demographic features, indications, and response rates of the patients that have used canakinumab.

**Methods:** This study was conducted in pediatric rheumatology clinic of Istanbul Kanuni Sultan Süleyman Research and Training Hospital. The files of the patients that canakinumab have been used between December 2012 and January 2017 were reviewed.

**Results:** Canakinumab was used in 23 patients. Diagnosis of the patients were; colchicine resistant (cr) FMF (15 cases), HIDS (2 cases), CAPS (2 FCAS cases and 1 CINCA case), sJIA (2 cases), and pyoderma gangrenosum (1 case). Canakinumab has been used for 24.8 months. Canakinumab was discontinued in 3 patients. In crFMF patients, the reason for use of biologic agent was resistance to colchicine that was described as having ≥3 attacks for 6 consecutive months despite maximal colchicine dose in this study. The crFMF patients had a striking female predominance; 13 girls and 2 boys. Mean age at the initial symptoms of FMF was 2.6 years. Twelve patients had M694V homozygous mutation, 1 patient had M694V/R761H compound heterozygous mutation and in 2 of the cases we could not have demonstrated a pathogenic mutation in the *MEFV* and other AID gene studies. In 10 of 15 crFMF patients, anakinra was used before canakinumab for 6.7 months. Mean number of attacks per year before colchicine was 18, after colchicine it was 6.8 and after biologic agent the number dropped to 0.7. At the time of enrollment, in 6 patients canakinumab was being used every month and in 8 patients every 3–4 months. Acute phase reactants regressed dramatically; CRP from 151.2 mg/L to 1 mg/L, SAA from 442.6 mg/L to 2.2 mg/L, ESR from 48 mm/h to 7 mm/h. Two of the HIDS cases of this cohort were siblings. Both had V377I mutation in the *MVK* gene. Anakinra was used for 9 months before canakinumab and canakinumab was being used for 3 months with resolution of the attacks. Two FCAS patients were using canakinumab for 5 and 9 months with total disappearance of rash. CINCA patient did not show dramatic response to IL-1 blockers with partial control of the acute phase reactants. Canakinumab 12 mg/kg/month with low dose prednisolone were used and laboratory and clinical parameters are under control. In one of the sJIA patients canakinumab was being used for 21 months with good response. But other sJIA case initially followed a polycyclic course and for 28 months canakinumab was used with a good response but later turned into polyarticular course under canakinumab treatment. A severe pyoderma gangrenosum patient had failed dapson and anakinra, and failed also canakinumab that is used for 9 months. We did not observe any side effect under canakinumab treatment.

**Conclusion:** In conclusion, canakinumab seem to be effective and safe in patients with AIDs.

**Disclosure of Interest**

None Declared

### B26 Efficacy of treatment using adalimumab in combination with methotrexate and methotrexate monotherapy in children with polyarticular Juvenile Idiopathic Arthritis and diagnostic meaning proinflammatory cytokins synovial fluids and blood

#### Vladimir Keltsev, Lyudmila Grebenkina

##### Rheumatology, Togliatti City Clinical Hospital #5, Togliatti, Russian Federation

###### **Correspondence:** Vladimir Keltsev

**Introduction:** Juvenile idiopathic arthritis (JIA) is the most common rheumatic disease in children and it is characterized by a primary lesion of the joints, organs and tissues with the formation of multiple organ failure of varying severity.

**Objectives:** The article describes the results of studying the efficacy and safety of adalimumab in combination with methotrexate (n = 14) and methotrexate monotherapy (n = 17) when treating the patients with polyarticular JIA refractory to the basic immunosuppressive therapy.

**Methods:** Retrospective analisis of 31 patient with JIA

**Results:** It was shown that the combination therapy induced the remission of arthritis in children with JIA in a shorter period of time. It should be noted that adalimumab in combination with methotrexate was well. tolerated and no serious adverse effects were recorded. Thus, the introduction of adalimumab in the treatment regimen of children with JIA refractory to the basic immunosuppressive therapy allowed for the rapid disease remission while preserving the effect in a significant number of patients during the following year.

**Conclusion:** Definition levels cytokins in blood and synovial fluids appears important investigation for diagnostic and effective therapy Juvenile Idiopathic Arthritis in patients.

**Disclosure of Interest**

None Declared

### B27 The clinical effectiveness of etanercept for treating Juvenile Idiopathic Arthritis in a single center experience

#### Kwang Nam Kim^1^, Jong Gyun Ahn^2^, Young Dae Kim^3^

##### ^1^Pediatrics, Hallym University Medical Center, AnYang, Korea, Republic Of; ^2^Pediatrics, Severance Children’s Hospital, Yonsei University, Seoul, Korea, Republic Of; ^3^Pediatrics, Inje University Paik Hospital, KoYang, Korea, Republic Of

###### **Correspondence:** Kwang Nam Kim

**Introduction:** JIA is a broad term that describes a clinically heterogeneous group of arthritis of unknown cause. Although none of the curative drug has been developed, the most important new drug has been introduced such as biologic products. Management of JIA is based on a combination therapy with MTX/etanercept recently.

**Objectives:** The aim of this study was to investigate efficacy of etanercept in JIA and to find out the difference efficacy of JIA subtypes. And the second aim is to announce the our results to the pediatrician who take care of JIA patients. Because there is few pediatric rheumatologist in Korea.

**Methods:** JIA patients (n = 53) who have been treated with etanercept during the 2010 through 2016 were chart reviewed retrospectively. Systemic JIA (n = 11). poly JIA (n = 16). extended olioarthritis (n = 25). ERA (n = 1). Mean age was 9 years (4-17years) and the sex ratio was 25:28 (boy : girl). We compaired the swollen joints and inflammatory markers every 3 months.

**Results:** The mean value of ESR and CRP were decreased within 3 months. Also the mean swollen joints were significantly decreased within 3 months. The adverse events were new onset or recurrent uveitis (n = 2). All of these two patients were switched to adalimumab.

**Conclusion:** The remarkable and rapid efficacy and the good safety profile of the etanercept. AntiTNF agents in children are usually well tolerated. RF (−) polyarticular JIA patients has best outcomes compared with other types. Systemic JIA have significantly improved, but mean value of ESR and CRP dose not normalized until 15 months. The pathophysiology of systemic JIA maybe different from that of other subtypes of JIA. However, physicians should remain alert for potential side-effects, especially reactivated tuberculosis during treatment with TNF inhibitors. All children should have a documented negative tuberculosis test before any biological therapy is started.

**Disclosure of Interest**

None Declared

## Vasculitides

### B28 Atypical Kawasaki disease with an unresolving pneumonia: a paediatric dilemma

#### Maria Cristina Maggio^1^, Rolando Cimaz^2^, Maria Concetta Failla^3^, Piera Dones^3^, Mirella Collura^4^, Giovanni Corsello^1^

##### ^1^University Department Pro.Sa.M.I. “G. D’Alessandro”, University of Palermo, Palermo, Italy; ^2^Pediatric Rheumatology Unit, Neurofarba Department, AOU Meyer, University of Florence, Florence, Italy; ^3^Paediatric Infectious Disease Unit, Children Hospital “G. Di Cristina”, ARNAS Palermo, Palermo, Italy; ^4^Unit of Paediatric Pneumology and Cystic Fibrosis, Children Hospital “G. Di Cristina”, ARNAS Palermo, Palermo, Italy

###### **Correspondence:** Maria Cristina Maggio

**Introduction:** Pneumonia is a possible associated radiological founding in Kawasaki disease (KD). However clinical impact is mild and the relieve is occasional, by chest radiogram. In a large series of chest radiograms in children with KD, about 15% of the patients had pathological pictures, mainly interstitial lung changes.

**Objectives:** Reports in the international literature on lung consolidation as part of KD or as the presenting symptom of KD are rare. Only a few cases of severe pneumonia in children affected by KD are reported.

**Methods:** A 2-year-old male was admitted to our unit with the presence of 5 days of fever, diffuse maculopapular eruption, conjunctivitis, lips cracking and erythema, bilateral conjunctivitis, oedema of hands and feet, cervical lymphadenopathy.

**Results:** He showed: CRP 16.24 mg/dl,, Hb 11.1, WBC count 23.790 (N: 65.5%), platelets 383.000, mild sterile pyuria. Echocardiography performed on the fifth day of the disease revealed the absence of coronary anomalies. The diagnosis was “typical KD” and he received IVIG (2 gr/kg) with ASA (50 mg/kg/day).

The child had been afebrile and showed reduction without complete resolution of the rash. A blood sample showed a significant reduction of CRP (6.09) and WBC (7.590; N: 37.4%): hence reduced the ASA dose was reduced. However fever started again 3 days later, in association with rash and conjunctivitis.

Blood sample evidenced: CRP: 9.08; WBC 16.850 (N: 69%)**;** platelet: 731.000; Na: 131; albumin: 3.2. He received a second dose of IVIG with resolution of fever, rash, conjunctivitis. ECG and echocardiography were still normal.

For the unexpected course of the disease, the chest radiographs were performed and revealed consolidation in the right middle lobe and opacity of the pulmonary hilum. TC confirmed a lung consolidation with pleural effusion. There were no respiratory symptoms or signs, and the respiratory rate, lung auscultation and oxygen saturation were normal.

He was treated with several antibiotics, including clarithromycin, ceftriaxone, chloramphenicol, imipenem and teicoplanin.

Quantiferon, bronchoalveolar lavage with culture and PCR were negative, excluding an infectious agent. 7 days later, ASA was reduced to the dose of 5 mg/kg, however fever restarted 48 hours later. The clinical worsening was associated with the following parameters: CRP: 10.24; WBC 14.100 (N: 69%)**;** platelet: 815.000. Chest ray evidenced a reduction of the pneumonia. The patient started methylprednisolone (2 mg/kg/day), associated with ASA (5 mg/kg/day). The clinical course showed the resolution of fever and the progressive normalization of CRP in 7 days. Chest ray and CT showed a progressive resolution of pneumonia. The tapering of methylprednisolone till the suspension and the resolution of the clinical signs was not associated with CAL.

**Conclusion:** Typical KD in pediatric patients can present with atypical manifestations that could make difficult the follow-up and create doubt in initial diagnosis, especially in non-responder patients. The clinical presentation of the child was uncommon. In fact he initially presented a complete KD however, when he had the relapses, the disease showed an unresolving pneumonia as the main manifestation of atypical KD.

In literature, the association of KD with unresolving pneumonia is rarely described: this case, however, showed a singular course: Kawasaki disease started as a “typical” form and the failure of IVIG treatment was linked to a complicated pneumonia. The pneumonia did not reveal an infectious aetiology and the child required steroids to resolve the disease. The real aetiology of pneumonia can explain the favourable course of the disease, after steroids were started. In conclusion, Atypical KD should be considered in the differential diagnosis of children with persistent fever and lobar consolidation unresponsive to antibiotics.

**Disclosure of Interest**

None Declared

### B29 Polyarteritis nodosa in children: nationwide survey of Japan

**Abstract withdrawn**

### B30 A case of childhood systemic polyarteritis nodosa as initial manifestation of digital gangrene

#### Jung-Woo Rhim^1^, Ki-Hwan Kim^1^, Soo-Young Lee^1^, Seung-Beom Han^1^, Jin-Han Kang^1^, Jae-Hee Chung^2^, Soo-Jung Lee^3^, Dae-Chul Jeong^1^

##### ^1^Pediatrics, College of Medicine, The Catholic University of Korea, Seoul, 3Pediatrics, International St. Mary’s Hospital, Catholic Kwandong University, Incheon, Korea, Republic Of; ^2^General Surgery, College of Medicine, The Catholic University of Korea, Seoul; ^3^Pediatrics, International St. Mary’s Hospital, Catholic Kwandong University, Incheon, Korea, Republic Of

###### **Correspondence:** Jung-Woo Rhim

**Introduction:** Childhood polyarteritis nodosa (PAN) is a rare systemic inflammatory disease characterized by systemic necrotizing vasculitis of the small or medium sized arteries that leads to infarct, and aneurysms in various organs.

**Objectives:** We wish to share our experience of systemic polyartertis nodosa.

**Methods:** Case presentation

**Results:** A 7 year-old girl was admitted due to sudden onset of painful blackish discoloration of right 2^nd^, 4^th^ and left 2^nd^ finger tips from proximal interphalangeal joints with fever. She could not walk by herself because of myalgia on both lower extremities. On admission, body temperature was 38.5 °C, and blood pressure 150/86. Physical examination showed gangrenous change of finger tips and tender swelling on right ankle joints. Initial laboratory data showed leukocytosis with dominant neutrophils, high C-reactive protein and ESR. Autoantibodies including ANA and ANCA showed negative. We identified microscopic hematuria. CT angiography for upper and lower extremities showed diffuse swelling with soft tissue fatty infiltration on both fingers, but no definite vessel and its branch abnormalities and renal arteries. Brain MRA showed luminal irregularity at intracranial major arteries compatible with arteritis, but no definite aneurysm. Electromyographic findings for motor showed decreased amplitudes in right median and ulnar nerves. Skin biopsy showed perivascular lymphohistiocytic infiltration compatible with vasculitis without any immunofluorescent staining. Culture for skin, blood, and urine was no any organisms. We diagnosed with systemic PAN including skin change, arthralgia and myalgia, hematuria, histologic findings and neuropathy. We started with low molecular weight heparin, prostaglandin E for prevention of thrombisis, and morphin for pain control. She received intravenous immunoglobulin and intravenous steroid. She was improved symptoms after steroid therapy, but still no changes of digital gangrene. We changed anti-coagulation therapy to Warfarin. She was tolerable after oral steroid and warfarin.

**Conclusion:** We present a girl with systemic polyarteritis nodosa as initial manifestation of digital gangrene.

**Disclosure of Interest**

None Declared

### B31 The experience in diagnostics and treatment of Kawasaki disease at Saint-Petersburg State Pediatric Medical University and Saint Petersburg Children’s Hospital №1

#### Andrei Santimov^1^, Regina Rupp^1^, Igor Alekseev^2^, Natalya Plutova^3^, Ekaterina Moskvina^3^, Marina Kruchina^3^, Aleksandra Tarasenko^3^, Natalya Sokolova^3^, Ekaterina Saveleva^3^, Ilia Bogdanov^3^, Dmitrii Ivanov^3^, Tatiana Kandrina^3^, Olga Kopanevich^3^, Anastasiia Grafskaia^3^, Natalia Ignateva^3^, Daria Pulukchu^1^, Natalia Pavlova^1^, Olga Kalashnikova^1^, Tatiana Kornishina^1^, Margarita Dubko^1^, Vyacheslav Chasnyk^1^, Mikhail Kostik^1^

##### ^1^Saint-Petersburg State Pediatric Medical University, Saint-Petersburg, Russian Federation; ^2^Saint Petersburg Children’s Hospital №1, Saint-Petersburg, Russian Federation; ^3^Saint Petersburg Children’s Hospital №1, Saint Petersburg, Russian Federation

###### **Correspondence:** Andrei Santimov

**Introduction:** Kawasaki disease (KD) is an acute systemic vasculitis of unknown etiology affecting most often children younger than 5 years of age and characterized by high fever, bilateral conjunctival hyperemia, mucosal changes of the oropharynx, erythematosus rash, erythema and indurative edema of the hands and feet, and cervical lymphadenopathy. Approximately 20–25% of untreated patients develop coronary artery changes with a range of severity from asymptomatic coronary artery dilatation or aneurysms to giant coronary artery aneurysms with thrombosis, myocardial infarction and sudden death. To date there is no official data on the incidence of KD in Russia. In Russia the disease to date is not enough known to a wider circle of doctors and often goes under the “mask” of other more common diseases. In Saint-Petersburg since 2010 has dramatically increased the recognition rate of KD.

**Objectives:** to review the experience of diagnosis and treatment of KD in the two largest hospitals in St. Petersburg.

**Methods:** the retrospective study included data on 30 children (18 boys, 12 girls) who were hospitalized with a diagnosis of KD in the clinics of Saint-Petersburg State Pediatric Medical University and Children’s Hospital No. 1 (St. Petersburg) from January 2011 to September 2016. Data is represented by the median and extreme ranges.

**Results:** the age of the children was 2,8 years (0,2; 4,6), 5 patients (16,7%) were under the age of 1 year. The children were hospitalized on the 5^th^ days of illness (1; 14 days), the diagnosis of KD was established on the 9^th^ day of the disease (3; 52). Immediately after diagnosis aspirin have got 27 children (90%). In the early stages (before 10 days of illness), therapy with intravenous immunoglobulin has received 15 children (50%), 1 of them received intravenous immunoglobulin before 5 days of illness (on day 3) and has not responded to the treatment. On 11–20 day (immediately after diagnosis) treatment with intravenous immunoglobulin received 10 children (33,3%), after which the fever decreased in all patients. Fever was stopped on day 11 (6; 23). Lesions in coronary arteries by ultrasound were diagnosed in 13 children (43,3%). The youngest patient who became ill at the age of 3 months and received intravenous immunoglobulin on the 30^th^ day of illness dead.

**Conclusion:** KD should always be included in the differential diagnosis in children of early age with prolonged fever, especially in children of the first year of life, which is often observed incomplete forms of KD. It is necessary to increase awareness among clinicians and doctors of ultrasonic diagnostics about KD.

**Disclosure of Interest**

None Declared

### B32 Isolated pulmonary artery involvement in childhood Takayasu arteritis

#### Shama Sowdagar^1^, Janani Sankar^1^, Venkateswari Ramesh^1^, Mahesh Janarthanan^2^

##### ^1^Department of Pediatrics, Kanchi Kamakoti Childs Trust Hospital, Chennai, India; ^2^Consultant Pediatric Rheumatologist, Kanchi Kamakoti Childs Trust Hospital, Chennai, India

###### **Correspondence:** Shama Sowdagar

**Introduction:** Takayasu arteritis is rare idiopathic chronic granulomatous inflammatory disease of the large vessels that involves the aorta or its branches and the pulmonary arteries.

**Objectives:** EULAR/PRINTO/PRES Ankara 2008 classification of childhood Takayasu arteritis requires angiographic abnormalities of the aorta or its main branches and pulmonary arteries showing aneurysm/dilatation (mandatory criterion) plus one of the five following criteria: Pulse deficit or claudication, four limbs BP discrepancy, bruits, hypertension and elevated acute phase reactant. In childhood Takayasu arteritis the thoracic and abdominal aorta are the most commonly involved vessels.

**Methods:** We describe the case of an 8 year old girl with Takayasu arteritis presenting with isolated pulmonary artery involvement.

**Results:** An 8 year old previously well girl presented with pyrexia of unknown origin of one month duration. She was initially evaluated at another centre where a routine chest X-Ray revealed dilated pulmonary vessels and patchy opacities in superior and lateral segments of right lower lobe and lingual. Though investigations were negative for tuberculosis she was started on empirical antituberculous therapy. In view of persistent fever, new onset cough and exertional dyspnoea she was referred to our centre.

On examination she had grade 2 clubbing, periorbital puffiness, elevated jugular venous pulse, loud second heart sound. Her 4 limb blood pressure readings were normal and there was no hypoxia.

Laboratory investigations showed anemia(9.3gms/dL), elevated inflammatory markers(CRP: 120 mg/L; ESR: 80 mm/hr). Chest x- ray showed marginal cardiomegaly with prominent pulmonary arteries. ECHO showed severe pulmonary hypertension and right ventricular dysfunction. cANCA was weakly positive and pANCA was negative. MRA chest and abdomen showed dilated main, right and left pulmonary arteries and focal thickening with narrowing in the distal right and left pulmonary arteries with dilatation of lobar pulmonary arteries. The thoracic and abdominal aorta were normal.Clinical history, laboratory parameters and imaging were suggestive of Takayasu arteritis with isolated pulmonary arterial involvement.

She was commenced on diltiazem and sildenafil for pulmonary hypertension. For the vasculitis she was treated with steroids, low dose aspirin and monthly cyclophosphamide.

**Conclusion:** Involvement of pulmonary arteries is common in Takayasu arteritis. Isolated pulmonary arteritis though rare, is reported in adult Takayasu disease. To our knowledge this is the youngest patient to present with isolated pulmonary artery involvement.

**Disclosure of Interest**

None Declared

### B33 Heart failure in childhood Takayasu arteritis: case report and literature review

#### Iulia E. Szabo^1^, Claudia Sirbe^2^, Cristina Pamfil^1,3^, Laura Damian^1^, Simona Rednic^1,3^

##### ^1^Department of Rheumatology, Emergency Clinical County Hospital, Cluj-Napoca, Romania; ^2^Department of Pediatrics, Emergency Clinical County Hospital, Cluj-Napoca, Romania; ^3^Department of Rheumatology, “Iuliu Hatieganu” University of Medicine and Pharmacy, Cluj-Napoca, Cluj-Napoca, Romania

###### **Correspondence:** Iulia E. Szabo

**Introduction:** Takayasu arteritis (TA) is a chronic granulomatous large vessels panarteritis predominantly affecting the aorta and its major branches. The diagnosis and treatment of childhood TA remains a challenge due to (1) differences in clinical spectrum compared to adult TA, (2) the invasive nature and radiation exposure of conventional angiography and (3) the paucity of available data regarding treatment options in childhood TA.

**Objectives:** The aim of this case report was to describe a single-center pediatric case of TA presenting with heart failure as the main disease manifestation and review four such cases reported in the literature.

**Methods:** PubMed, MEDLINE and Google Scholar were screened using the following key words: “Takayasu”, “Takayasu arteritis”, “heart failure”, “cardiomyopathy”, “dilated cardiomyopathy”. After applying age, language, article type and text availability filters, four articles were included in this literature review. Several parameters were analyzed: age at diagnosis, gender, left ventricular ejection fraction (LVEF) on echocardiography, vascular imaging techniques, glucocorticoid regime, immunosuppressive agents, biological therapy, surgical interventions, angioplasty and response to medical or surgical treatment.

**Results:** A 14-year-old girl presented with signs and symptoms of acute heart failure. Past medical history revealed progressive dyspnea on exertion, cough and claudication of the lower limbs during the past year. She was repeatedly treated with antibiotics for community-acquired pneumonia. Physical examination showed high, discordant blood pressure in the upper limbs (right arm 190/110 mmHg, left arm 150/80 mmHg), no peripheral arterial pulses in the lower and left upper limbs, basal crackles in both lungs, hepatosplenomegaly and lower limb edema. Ecocardiography and cardiac magnetic resonance identified severe left ventricular dilation, diffuse hypokinesia and LVEF of 23%. Magnetic resonance angiography revealed 75% stenosis of the left common carotid artery, complete occlusion of the left subclavian artery and 80% stenosis of the descending thoracic aorta. According to the 2010 EULAR/PRINTO/PRESS classification criteria the diagnosis of TA was established and induction therapy with pulse methylprednisolone in combination with cyclophosphamide (0.5-1 g/m2/month, for 6 months) was initiated followed by maintenance therapy with oral corticosteroids (prednisone 1.6 mg/kg/day). Poor clinical and echocardiographic improvement was obtained (LVEF 30%) and unfortunately the patient was lost to follow-up. Database literature search identified four cases of heart failure as chief manifestation of childhood TA. All patients were females. Ecocardiography showed decreased LVEF in all cases, with a mean LVEF of 28%. The most common vascular anomaly identified on computed tomography or magnetic resonance angiography was severe stenosis or occlusion of the descending aorta. Angiography was available in two patients and revealed a Numano’s type III TA. Two patients benefited from transcatheter stent implantation with marked improvement of the cardiac function. One patient underwent bypass grafting but was lost to follow-up. Treatment with Tocilizumab 8 mg/kg/monthly proved to be effective and safe in one case report.

**Conclusion:** Severe stenosis of the thoracic aorta leading to heart failure in children is rare and difficult to treat. In these cases glucocorticoids and conventional immunosuppressive agents alone are not able to induce remission and improve cardiac function. More aggressive strategies such as surgical interventions, angioplasty and biological therapy have shown to be superior in preserving heart function.

**Disclosure of Interest**

None Declared

## Scleroderma and related syndromes

### B34 Localized juvenile extensive scleroderma and systemic lupus erythematosus

#### Maria Deac^1^, Cristina Pamfil^2,3^, Mihaela Sparchez^4^, Ileana Filipescu^1,2^, Mirela Parvu^5^, Dumitrita Balint^6^, Ancuta Nicoara^1^, Simona Rednic^1,2,7^, Laura Damian^1,8^

##### ^1^Emergency Clinical County Hospital, Cluj-Napoca, Romania; ^2^Rheumatology, Iuliu Hatieganu University of Medicine and Pharmacy, Cluj-Napoca, Romania; ^3^Centre for Rare Musculoskeletal Autoimmune and Autoinflammatory Diseases, Cluj-Napoca, Romania; ^4^Pediatrics, Iuliu Hatieganu University of Medicine and Pharmacy, Cluj-Napoca, Romania; ^5^Rheumatology, Emergency Clinical County Hospital, Targu-Mures, Romania; ^6^Emergency Clinical County Hospital, Targu-Mures, Romania; ^7^Centre for Rare Musculoskeletal Autoimmune and Autoinflammatory Diseases, Cluj-Napoca, Romania; ^8^Rheumatology, Centre for Rare Musculoskeletal Autoimmune and Autoinflammatory Diseases, Cluj-Napoca, Romania

###### **Correspondence:** Cristina Pamfil

**Introduction:** Juvenile morphea is not always a dermatological disease only of aestethic concern, as it may be associated with systemic diseases.

**Objectives:** To present a case of systemic lupus erythematosus (SLE) in a patient with an extensive morphea in the early childhood.

**Methods:** A 27-year patient, with an extensive morphea at the age of 6 and a SLE diagnosed at the age of 18 years, was referred to our tertiary unit for altered clinical status and the assessment of recent-onset arterial hypertension and right lower limb swelling.

**Results:** Clinical examination and further investigations revealed polyserositis including pericarditis, hepatosplenomegaly and no deep venous thrombosis. Laboratory showed antinuclear antibodies with anti-dsDNA, positive anti-Scl-70 antibodies, C3 and C4 consumption, anemia (7.8 g/dL), thrombocytopenia (65 000 Plt/mmc), increased D-dimers and a nephrotic-range proteinuria (6 g/24 hours). A higher ferritin (675 mg/dL, normal < 450 mg/dL) and rare schistocytes on the blood smear were noted. The videocapillaroscopy did not reveal megacapillaries. The clinical picture of the nephrotic syndrome was modified by the apparently normal-looking skin (except from a slight hyperpygmentation) involving the whole left lower limb, actually not distensible due to the former morphea. She was anticoagulated, treated with cyclophosphamide (Eurolupus scheme), hydroxychloroquine, low-dose prednisone, angiotensin-converting enzyme inhibitors and albumin, with slow improvement. Two months later, before the end of the initial cure, an initial unplanned pregnancy was lost. After azathioprine, hydroxychloroquine and fraxyparine, she successfully conceived a healthy babygirl, born at term, without complications for the mother. She is currently well, on azathioprine, hydroxychloroquine, low-dose aspirin and vitamin D.

**Conclusion:** Lower-limb morphea in childhood may modify the clinical appearance of a bilateral edematous syndrome, even when the skin is not much modified. Localized scleroderma may be accompanied by anti-Scl70 antibodies, even in the absence of a full systemic sclerosis picture. Informed consent to publish has been obtained from the patient/parent/guardian.

Funding: PN-II-RU-TE-2014-4-2708

**Disclosure of Interest**

None Declared

